# Natural Antifungal Alkaloids for Crop Protection: An Overview of the Latest Synthetic Approaches

**DOI:** 10.3390/ph18040589

**Published:** 2025-04-18

**Authors:** Denise Dozio, Francesca Sacchi, Andrea Pinto, Sabrina Dallavalle, Francesca Annunziata, Salvatore Princiotto

**Affiliations:** Department of Food, Environmental and Nutritional Sciences (DeFENS), University of Milan, via Celoria 2, 20133 Milan, Italy; denise.dozio@unimi.it (D.D.); francesca.sacchi@unimi.it (F.S.); andrea.pinto@unimi.it (A.P.); sabrina.dallavalle@unimi.it (S.D.)

**Keywords:** alkaloids, crop protection, fungicide, total synthesis, phytopathogenic fungi

## Abstract

Alkaloids are nitrogen-containing compounds naturally occurring in plants, microorganisms, and marine organisms. Potent biological activities have been reported to date, ranging from neuroprotective to antioxidant and anticancer effects. Alkaloids have recently gained attention as potential antifungal agents for crop protection due to their broad spectrum of activity, eco-friendly nature, and ability to overcome some of the issues associated with synthetic fungicides, such as resistance development and environmental contamination. Several efforts have been made to obtain natural and nature-derived alkaloids endowed with significant activity against numerous pathogenic fungal strains. In this review, we collect synthetic strategies developed over the past decade to produce alkaloid fungicides for crop protection. Special emphasis is given to recent advancements in obtaining pure natural compounds and more potent analogs endowed with tailored and optimized properties.

## 1. Introduction

Globally, 2.4 billion people struggle daily with food insecurity, facing either moderate or severe shortages that impact their lives and safety. The global population is expected to grow by approximately 30% by 2050, increasing food insecurity and limiting the possibility of achieving the zero-hunger goal established by the United Nations in 2015 [1]. Agriculture plays a pivotal role in food waste, recording losses both in pre-and post-harvest handling, mainly because of the heavy impact of plant diseases. Of these, pathogenic fungi infections alone are responsible for 70–80% of the world’s major crop devastations and are considered a serious threat to global food security [2].

Fungicides are one of the most valuable means to combat this emergency. However, their efficiency in dealing with the insurgence of infections in intensive cultivation is slowed by the development of resistant strains, improper crop rotations, the presence of spores in the soil, and monocultures [3,4].

Additionally, the use of synthetic agrochemicals is raising strong concerns among consumers and researchers due to their hazards to the environment, mainly because of their slow turnover and their long-lasting toxic remains. For example, lipophilic residues can be detected in both water and soil, damaging the non-target animals and plants, as they act on basic biological processes not specific to fungi (e.g., energy production) [3]. The health hazards to humans, related to the use of pesticides, are also worth mentioning, as they are considered responsible for an increased risk of developing cancers and cardiovascular, neurological, and endocrine-related diseases [5]. Consequently, in recent years, there has been increased attention paid to the replacement of synthetic agrochemicals with safer molecules, such as natural and nature-derived compounds.

Natural products (NPs) represent between 30% and 35% of the drugs approved by the FDA over the last 35 years. The high structural diversity, specificity, and novel mode of action of natural bioactive compounds make nature an endless source of suitable drug candidates. These are usually secondary metabolites produced in low amounts in response to biotic or abiotic stress conditions, and their composition can vary due to the environment or the sourcing season [6]. Moreover, since these compounds could be bio-transformed by plants and microorganisms into safe metabolites, they usually display a more rapid turnover and a subsequent mild impact on the environment.

Alkaloids are a heterogeneous and wide class of compounds which are characterized by the presence of at least one atom of nitrogen. They are primarily derived from plants, fungi, and some marine organisms [7]. Their pharmacological properties have been deeply studied over the last centuries, starting with the well-known example of morphine [8]. Neuroprotective effects, as well as antioxidant, antimicrobial, and anticancer properties, are only some of the bioactivities ascribed to these compounds [9,10,11]. In particular, antiviral, antibacterial, and antifungal activities have been reported; moreover, in recent years, their potential as a treatment for phytopathogenic fungi has raised interest among the scientific community [12,13].

In the framework of crop protection, alkaloids exhibit a broad spectrum of activities against different fungal species, including those responsible for plant diseases, such as *Fusarium*, *Botrytis*, and *Alternaria*. Additionally, with the rising concerns about the environmental impact of synthetic fungicides, alkaloids offer a more eco-friendly alternative. Many of them degrade naturally in the environment, as they are taken up and accumulated by acceptor plants, thus reducing the risk of soil and water contamination [14]. Finally, alkaloids may reduce the likelihood of resistance development in fungal populations, thanks to their diverse chemical structures and modes of action (e.g., inhibition of cell wall synthesis, disruption of membrane integrity, inhibition of enzymes necessary for fungal metabolism, and DNA damage).

The interest in developing synthetic approaches to attain bioactive natural alkaloids is widely observed in the literature. This is mainly due to the difficulty of extracting them from their natural matrices, driving up the cost and effort needed to gather the compounds of interest in reasonable yields and purities. As a matter of fact, alkaloid availability is often constrained by factors like specific plant cultivation conditions, slow growth rates, and difficulties in large-scale harvesting. Under these circumstances, chemical preparation offers a more controlled, scalable, and cost-effective method of production, enabling the obtainment of alkaloids in the necessary quantities to carry out bioactivity studies and potentially, effective crop protection. Moreover, synthetic approaches open the door to explore structure–activity relationships (SAR), enabling structural modifications of selected alkaloids to improve their effectiveness against fungal pathogens while minimizing negative impacts on non-target organisms. In this way, alkaloid analogs and derivatives can be tailored to produce desirable properties, such as increased solubility, enhanced stability against environmental stressors (e.g., heat and light), and more efficient delivery to target fungi.

**Figure 1:**
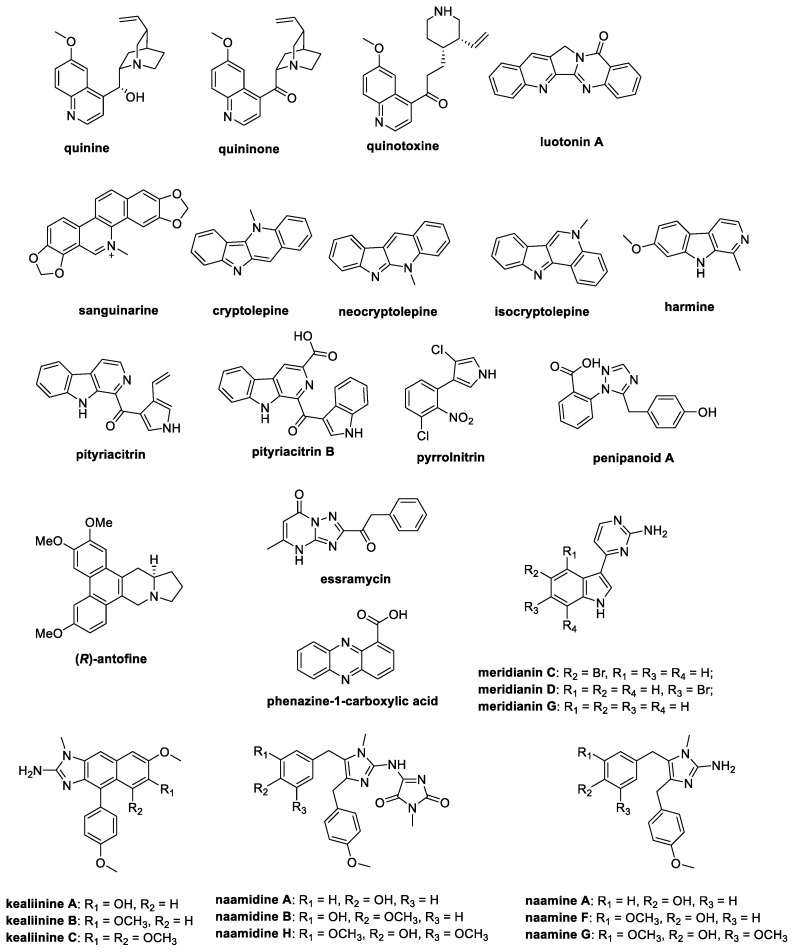
Chemical structures of the natural antifungal alkaloids whose synthesis is reported in this paper.

This review aims to perfuse the literature from the last ten years to obtain information regarding the total synthesis of natural alkaloids with antifungal activity in the field of crop protection, exploiting the SciFinder^®^, PubMed, and Scopus databases. Derivatives and analogs have been wittingly excluded from this work to highlight the variety and potential of natural parent compounds. For this reason, the attention will focus on the most investigated classes of alkaloids as antifungal agents, i.e., quinoline-, isoquinoline-, and indole-containing alkaloids, together with substituted indoles, imidazoles, and pyrroles, with the major purpose of supporting research groups working on the synthesis and investigation of such chemotypes as crop protectors (Figure 1).

## 2. Quinoline- and Isoquinoline-Containing Alkaloids

### 2.1. Quinine and Quinine Analogs (from Plants)

**Figure 2:**
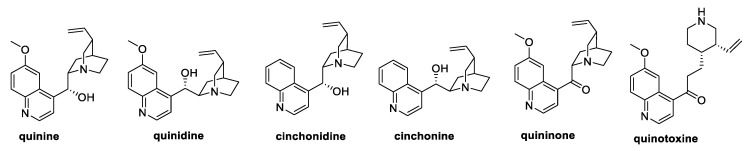
Structure of quinine and its natural analogs.

Among the natural quinoline alkaloids, quinine (Figure 2) is undeniably one of the most studied. Isolated from the bark of several species of *Cinchona* and *Remijia* trees, quinine shows several biological activities, ranging from antimalarial, anti-inflammatory, and anticancer to antifungal types [15,16]. Specifically, quinine was reported to inhibit mycelia growth of *F. graminearum* by 46%, whereas a 30% inhibition was observed against *P. zeae*, *R. solani*, *M. melonis*, and *M. oryzae* [15].

Several natural quinine analogs, like quinidine, cinchonidine, cinchonine, quininone, and quinotoxine (Figure 1), were also evaluated as antifungal agents against pathogenic fungi, showing weak antifungal activities at 100 µg/mL concentration [15].

**Scheme 1:**
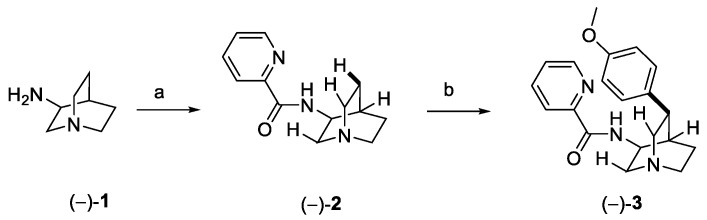
C(sp^3^)–H activation of 3-aminoquinuclidine and synthesis of intermediate **3**: (a) picolinic acid, CDI, DMF; (b) 4-iodoanisole, Pd(OAc)_2_, Ag_2_CO_3_, DMF.

The stereoselective total synthesis of quinine was reported for the first time in 2001 in 15 steps, starting from enantiomerically pure (*S*)-4-vinyl butyrolactone [17], while several new synthetic routes have been developed to obtain this relevant compound over the last decades [18,19]. In 2018, O’Donovan et al. reported a novel approach based on two stereoselective key steps: (1) a C–H activation followed by (2) an aldol reaction, in order to connect the two heterocyclic moieties of quinine [20]. The C(sp^3^)-H activation (Scheme 1) was accomplished by treating (−)-3-aminoquinuclidine (**1**) with 2-picolinic acid to obtain compound (−)-**2**, with the amide fragment acting as the directing group towards the selective activation of the H at C(5). The Pd-catalyzed formation of a new C(sp^3^)–C(sp^2^) bond with the anisole fragment allowed the obtainment of enantiomerically pure (−)-**3** with complete regio- and stereoselectivity.

**Scheme 2:**
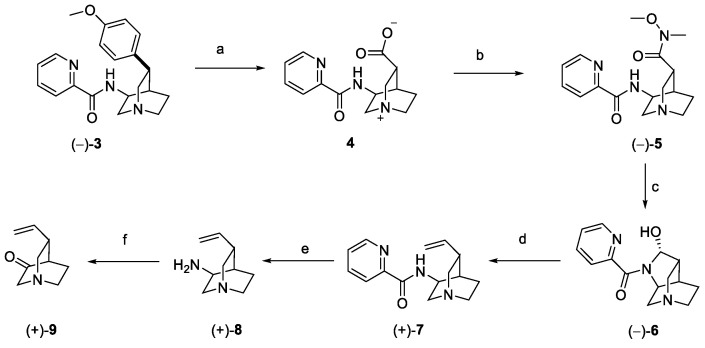
Synthesis of building block **9**. (a) RuCl_3_, NaIO_4_, H_2_O, EtOAc, CH_3_CN; (b) HATU, MeNHOMe·HCl, Et_3_N, DMF; (c) DIBAL-H, DCM; (d) Ph_3_PMeBr, LiHMDS, DMSO, THF; (e) Zn, HCl, Zn(OTf)_2_, H_2_O; (f) IBX, *p*-TsOH, CH_3_CN.

Intermediate **3** underwent oxidative degradation of the benzyl moiety under ruthenium catalysis, leading to zwitterion carboxylic acid **4** (Scheme 2). Generation of the Weinreb amide **5**, followed by DIBAL-H mediated reduction, yielded optically pure hemiaminal **6**. Wittig olefination with methylenetriphenylphosphorane afforded alkenyl-quinuclidine **7**, further converted to the free amine **8** under reducing conditions. Final oxidation in the presence of IBX generated optically pure **9**, as the key building block for the synthesis of quinine [20].

**Scheme 3:**
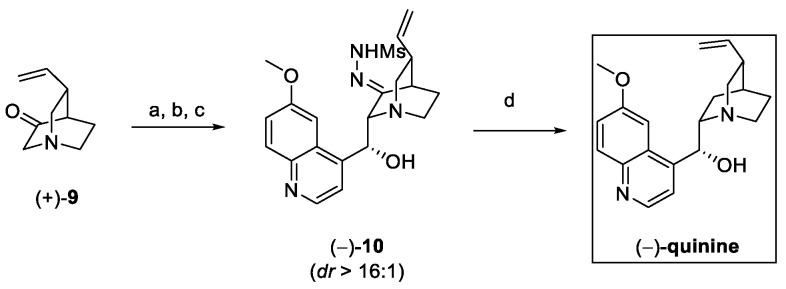
Conversion of (+)-**9** to (−)-quinine. (a) LiHMDS, THF; (b) 6-methoxyquinoline-4-carbaldehyde; (c) Ti(O-*i*Pr)_3_Cl, MsNHNH_2_; (d) LiAlH_4_, MeOH, THF.

Aldol reaction between **9** and the suitable quinoline carbaldehyde was followed by the in situ conversion of the ketone to sulfonylhydrazone (**10**). Under these conditions, epimerization-free reduction in presence of LiAlH_4_ takes place, affording (−)-quinine in 10 steps, with a 5% overall yield (Scheme 3).

**Scheme 4:**
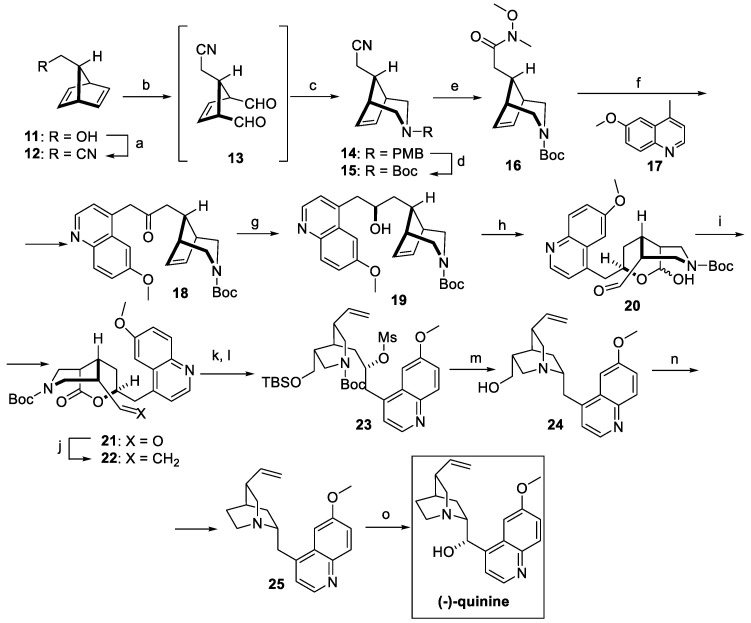
Total synthesis of (−)-quinine starting from intermediate **11**: (a) acetone cyanohydrin, PPh_3_, DEAD, Et_2_O; (b) OsO_4_, NMO, acetone/H_2_O, then Pb(OAc)_4_, DCM; (c) NaBH_3_CN, PMBNH_2_, AcOH, MeOH; (d) triphosgene, Et_3_N, DCM, then NaHCO_3_, Boc_2_O; (e) NaOH/MeOH, then EDC, HOBt, Et_3_N, MeNH(OMe)•HCl, DCM; (f) **17**, LDA, THF; (g) NaBH_4_, MeOH; (h) OsO_4_, NaIO_4_, 2,6-lutidine, dioxane/H_2_O; (i) DMP, DCM; (j) MeSO_2_PT, KHMDS, THF; (k) LiAlH_4_, THF; (l) TBSOTf, 2,6-lutidine, DCM, then MsCl, Et_3_N, DCM; (m) DCM/TFA, then NaHCO_3_, MeCN; (n) (COCl)_2_, DMSO, Et_3_N, DCM, then [Rh(COD)Cl]_2_, dppp, diglyme; (o) NaH/DMSO, then O_2_.

A “local desymmetrization” approach was exploited by Lee and Chen for the synthesis of pure quinine and quinidine, both obtained starting from the same racemic intermediate (Scheme 4) [21]. Commercially available primary alcohol **11** was converted to nitrile **12** under Mitsunobu conditions. The resulting diene was dihydroxylated and underwent oxidative cleavage to afford dialdehyde **13**. Ring closing by reductive amination yielded the protected piperidine fused bicyclic **14**, which was converted to carbamate **15** and then to Weinreb amide **16**. The quinoline fragment was introduced by reaction with 6-methoxylepidine **17** in the presence of LDA (compound **18**), which was further reduced to racemic alcohol **19**. Functionalization of the endocyclic double bond resulted in a mixture of hemiacetals **20**, further oxidated with DMP as the key desymmetrization step to obtain lactone **21** as a single stereoisomer, then converted to the vinyl substituent under modified Julia conditions (compound **22**). Ketone reduction and conversion to mesylate **23** allowed for, upon Boc deprotection, the nucleophilic substitution and formation of the characteristic quinuclidine moiety (compound **24**). Swern oxidation of the primary alcohol resulted in the corresponding aldehyde, which underwent Rh-catalyzed deformylation to dehydroxyquinine **25**, which was selectively hydroxylated to natural quinine, obtained with an overall yield of 17% [21].

**Scheme 5:**
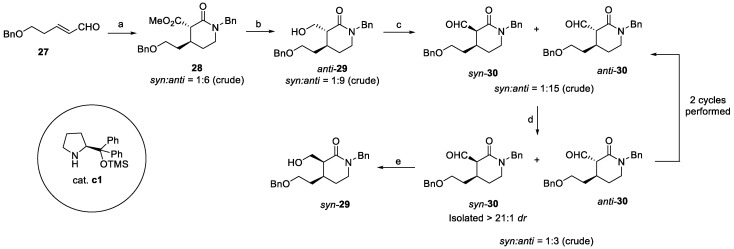
Synthesis of key intermediate *syn*-**29**: (a) dimethyl malonate, 10 mol% **c1**, EtOH, then BnNH_2_, NaBH(OAc)_3_, DCM, 97% *ee*, 85%; (b) LiAlH_4_, THF, >21:1 *dr* (isolated), 78%; (c) (COCl)_2_, DMSO, DCM, then TEA, 9:1 *dr* (isolated), 82%; (d) pyrrolidine, AcOH, DCM; (e) NaBH_4_, MeOH.

Lastly, Jiang et al. synthetized quinine via catalytic enantioselective cascade transformations [22]. In this case, starting from α,β-unsaturated aldehyde **27**, a one-pot cascade in the presence of dimethyl malonate led to the α-substituted lactam *anti*-**28**, with 6:1 *dr* and 97% *ee* (Scheme 5). Reduction to primary alcohol afforded *anti*-**29**, which was further oxidized to aldehyde **30**. Mild piperidine-catalyzed epimerization allowed for the isolation of *syn*-**30** with >21:1 *dr*, which was ultimately reduced to the desired *syn*-**29** [22].

**Scheme 6:**
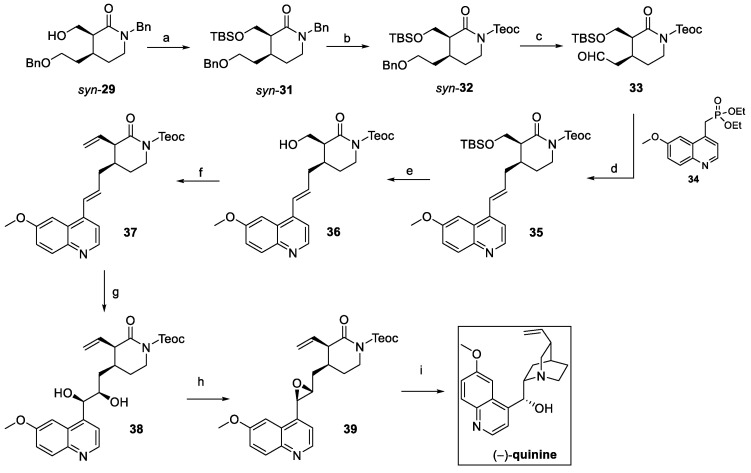
Total synthesis of (–)-quinine starting from intermediate isolated stereoisomer *syn*-**29**: (a) TBSCl, imidazole, dry DMF; (b) TMSCH_2_CH_2_OH, triphosgene, K_2_CO_3_, toluene; (c) i. Pd/C, H_2_, MeOH, ii. (COCl)_2_, DMSO, DCM, then TEA; (d) **34**, NaH, THF; (e) HCl/MeOH; (f) i. (COCl)_2_, DMSO, DCM, then TEA; ii. Ph_3_P^+^CH_3_Br^–^, KO*t*-Bu, THF; (g) ADmix-β, CH_3_SO_2_NH_2_, *t*-BuOH, H_2_O, >21:1 *dr*, 82%; (h) i. trimethylorthoacetate, PPTS, DCM; ii. TMSCl, DCM; iii. K_2_CO_3_, MeOH; (i) CsF, *t*-BuOH, DMF.

Such an approach was crucial to guarantee the required stereochemical control so that the debenzylation and further oxidation of suitably protected *syn*-**31** could afford aldehyde **33** (Scheme 6). The Horner–Emmons reaction with the phosphonated quinoline **34** yielded olefin **35**, treated to obtain the vinyl key intermediate **37**. Catalytic chemo- and enantioselective dihydroxylation of the internal double bond using AD-mix-*β* gave diol **38**, as a precursor of epoxide **39**. Final conversion of the lactam ring to piperidine and the deprotection of the TEOC resulted in the stereoselective intramolecular cyclization on the epoxide, affording (−)-quinine in 20% overall yield from *syn*-**29** [22].

**Scheme 7:**
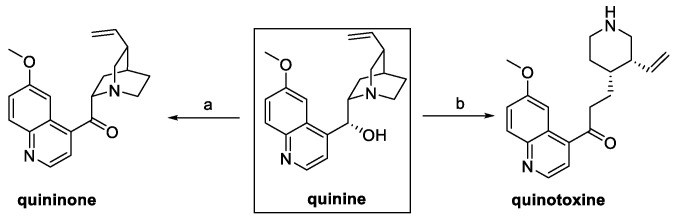
Synthesis of quininone and quinotoxine starting from quinine. (a) *t*-BuOK, benzophenone, toluene; (b) H_2_O/AcOH.

Among the natural quinine analogs, quininone and quinotoxine can be considered as derivatives of quinine itself. In fact, oxidation of the secondary alcohol in the presence of potassium *tert*-butoxide and benzophenone yielded quininone, whereas the boiling aqueous acetic acid treatment led to the quinuclidine bicyclic opening and hydroxyl group oxidation to quinotoxine (Scheme 7) [15].

### 2.2. Luotonin A (from Plants)

**Figure 3:**
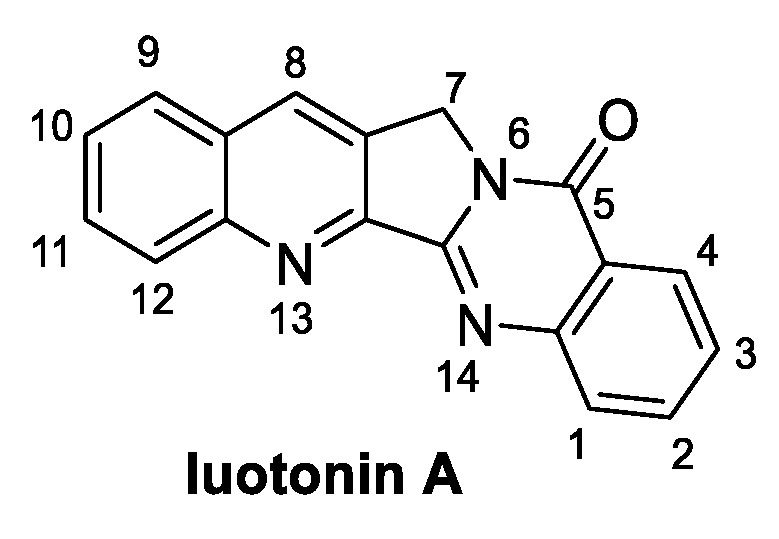
Chemical structure of luotonin A.

Luotonin A (Figure 3) is a quinoline alkaloid extracted from the Chinese medicinal plant *Peganum nigellastrum*. It specifically targets DNA topoisomerase I, resulting in a potential antitumoral natural compound [23]. Notably, evaluation of the antifungal activity of luotonin A resulted in an EC_50_ value of 0.50 mM against *B. cinerea* and *M. oryzae* [24]. Examples of its total synthesis have been reported at the end of the 20th century; however, over the last decade, several authors have proposed alternative synthetic routes, as discussed hereafter.

**Scheme 8:**
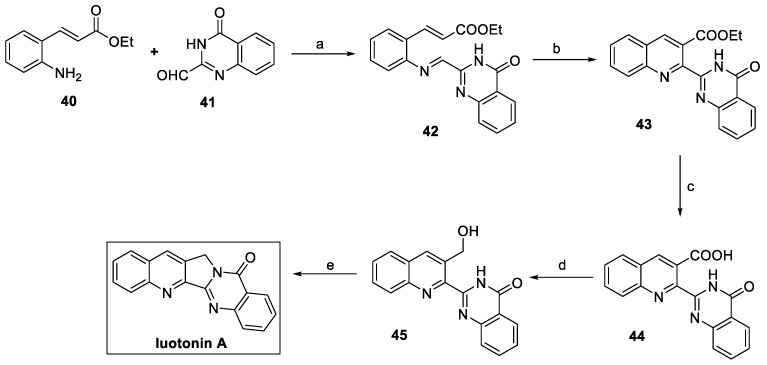
Synthesis of luotonin A. (a) MgSO_4_, Na_2_SO_4_, THF; (b) 1,2,4-trichlorobenzene, μW; (c) KOH, MeOH/H_2_O; (d) i. (COCl)_2_, DCM, DMSO; ii. NaBH_4_, THF; (e) PPh_3_, DIAD, THF.

In 2016, Kwon et al. described the total synthesis of luotonin A via the condensation between 2-amino cinnamate **40** and quinazolinone-2-carbaldehyde **41** (Scheme 8). The resulting aldimine **42**, in this case, acts as a direct precursor of the fused pentacyclic moiety. Thermal electrocyclization under microwave irradiation at 160 °C generated the desired quinoline core (compound **43**). Suitable functionalization of the ester moiety yielded alcohol **45**, which finally underwent an intramolecular Mitsunobu reaction to the desired natural product luotonin A, with an overall yield of 32% in five steps [25].

**Scheme 9:**
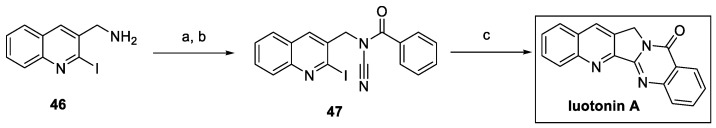
Synthesis of luotonin A by copper-catalyzed photoinduced radical domino cyclization reaction. (a) BrCN, Na_2_CO_3_, THF, then (b) BzCl, NaH, THF; (c) [(DPEphos)(bcp)Cu]PF_6_, Cy_2_NiBu, K_2_CO_3_, light (420 nm), MeCN.

The synthesis of luotonin A was carried out by Baguia et al. in the framework of a copper-catalyzed photoinduced radical domino cyclization of ynamides and cyanamides [26]. In particular, aminomethylquinoline **46** was converted to *N*-benzoylcyanamide **47** by cyanation and benzoylation reactions (Scheme 9). The photocatalyzed cyclization of **47** led to the quinazoline core and the fused pentacyclic system of luotonin A by 5-*exo*-dig and 6-*endo*-trig consecutive cyclizations.

### 2.3. Sanguinarine (from Plants)

**Figure 4:**
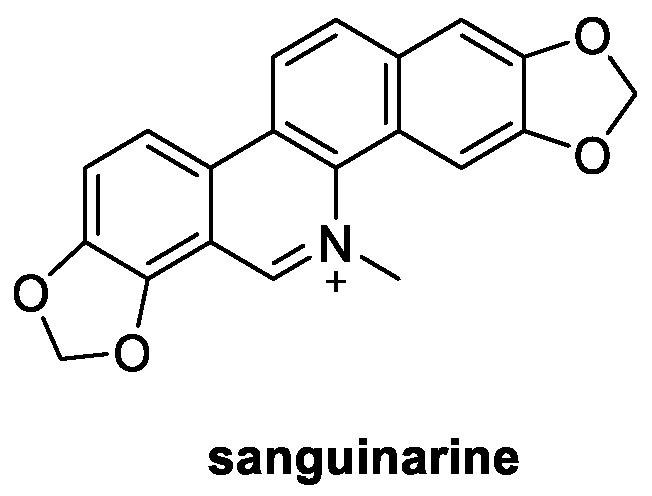
Chemical structure of sanguinarine.

Extracted from the rhizomes of the plant species *Sanguinaria canadensis*, isoquinoline alkaloid sanguinarine (Figure 4) is present in nature both as a charged iminium (pH 2–6) or an uncharged alkanolamine (pH 6.5–9.0) [27]. From the biological point of view, sanguinarine is well known not only for its remarkable antitumoral effect but also for its antioxidant and antifungal activities [28,29]. In particular, the greatest inhibitory activity was observed against *M. oryzae* (EC_50_ = 6.96 μg/mL), which was even better than that of the positive control azoxystrobin (EC_50_ = 12.04 μg/mL). Indeed, treatment of *M. oryzae* mycelia with sanguinarine at 10 μg/mL induced morphology and cell membrane integrity damage. Furthermore, the reactive oxygen species production, mitochondrial membrane potential, and nuclear morphometry of the mycelia were changed, and the membrane function and cell proliferation of the mycelia were destroyed. Moreover, sanguinarine significantly suppressed the spore germination process in *M. oryzae* at the concentration of 50 μg/mL [29].

**Scheme 10:**
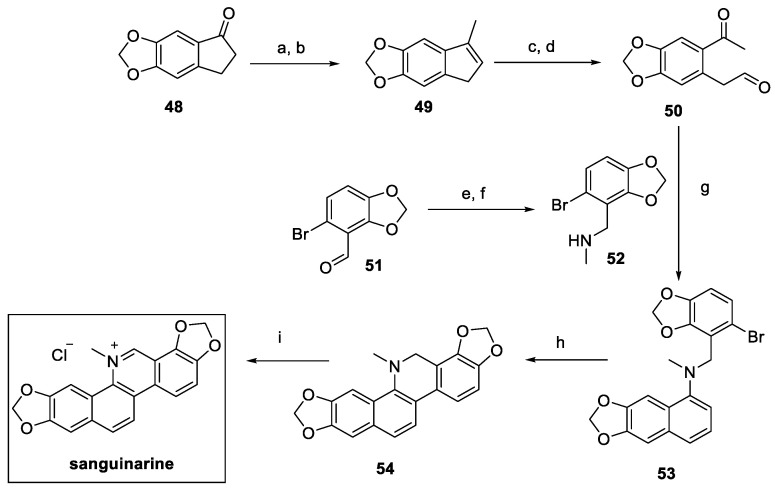
Synthesis of sanguinarine chloride salt. (a) MeLi, CeCl_3_, THF, then (b) PPTS, DCM; (c) K_2_OsO_2_(OH)_4_, NMO, THF/H_2_O then (d) NaIO_4_·SiO_2_, Et_2_O; (e) MeNH_2_, EtOH, then (f) NaBH_4_, MeOH, quant; (g) ZnCl_2_, DCE; (h) phenanthroline, *t*-BuOK, benzene; (i) DDQ, NaOH, benzene then HCl.

The total synthesis of sanguinarine was carried out by Tatton in 2014, starting from commercially available indanone **48**. The introduction of the methyl group via methyllithium and further dehydration (compound **49**, Scheme 10) was essential to guarantee the oxidative cleavage to **50**, followed by the enamine-mediated cyclization to naphthylamine **53**. Intramolecular substitution yielded the dihydro isoquinoline core in **54**, oxidized by DDQ and treated with HCl to afford the desired natural product sanguinarine in the form of chloride salt [30].

### 2.4. Cryptolepine, Neocryptolepine, and Isocryptolepine (from Plants)

**Figure 5:**
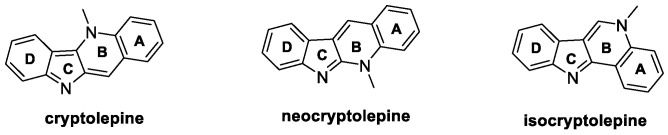
Chemical structures of natural cryptolepine, neocryptolepine, and isocryptolepine.

Cryptolepine, neocryptolepine, and isocryptolepine (Figure 5) are isomeric indoloquinoline alkaloids isolated from the roots of *Cryptolepis sanguinolenta* (*Periploaceae*), a tropical shrub indigenous to West Africa, consisting of four fused rings (A–D, Figure 5) [31]. As for their biological potential, cryptolepine effectively inhibits *S. sclerotiorum* (EC_50_ = 5.507 μg/mL) and *B. cinerea* growth (EC_50_ = 0.050 μg/mL), whereas neocryptolepine is active against *R. solani*, both in vitro and in vivo. Shang et al. demonstrated that neocryptolepine can inhibit complex III by binding UQCRFS1 (cytochrome b-c1 complex subunit Rieske) protein, blocking the ion transfer, and affecting oxidative phosphorylation, ultimately leading to mycelium death [32]. In addition, neocryptolepine significantly inhibits sclerotia formation in *R. solani* [33]. Isocryptolepine shows moderate antifungal activity against *F. graminearum* and *B. cinerea,* with an inhibition of mycelia growth of 34% and 46%, respectively, at 50 μg/mL [34]. The total synthesis of the three natural alkaloids, along with the efforts aimed at their structural modifications over the last ten years, have been collected and are hereafter reported.

#### 2.4.1. Cryptolepine

**Scheme 11:**
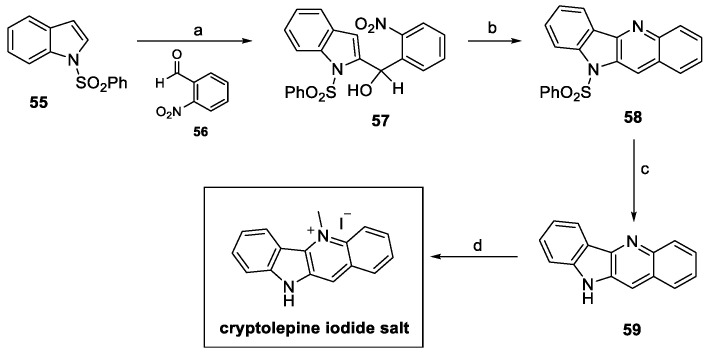
Synthesis of cryptolepine iodine salt. (a) *n*-BuLi, THF, then **56**; (b) PPh_3_, MoO_2_Cl_2_(dmf)_2_, toluene; (c) aq. NaOH, MeOH; (d) CH_3_I, sulfolane.

In 2016, Parvatkar and Parameswaran proposed a four-step pathway for the synthesis of cryptolepine salts, using a tandem reductive cyclization–dehydration protocol, as illustrated in Scheme 11 [31]. The C-2 lithiation of commercially available *N*-sulfonylindole **55** with *n*-BuLi allowed for functionalization with suitable benzaldehyde **56** to provide the corresponding 2-substituted indole derivative **57**. Mo-catalyzed reductive cyclization and dehydration resulted in the formation of tetracyclic **58**. Indole deprotection under alkaline conditions afforded compound **59**, while chemoselective methylation at the pyridine nitrogen using methyl iodide in sulfolane provided the desired cryptolepine salt in a 17% yield over four steps [31].

**Scheme 12:**
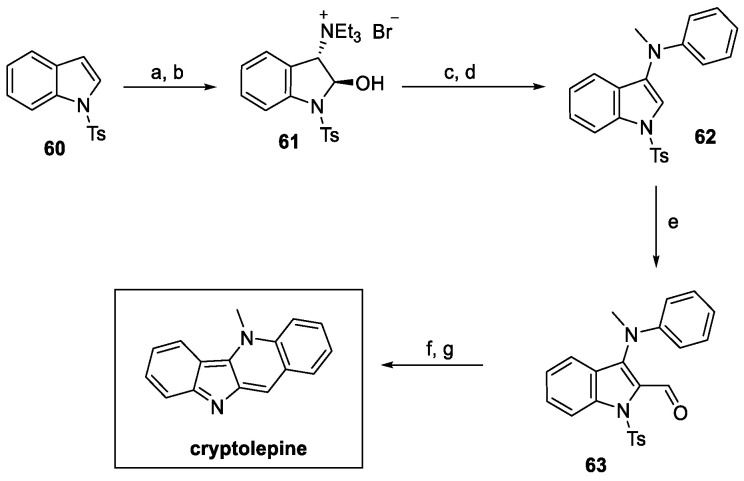
Synthesis of cryptolepine, starting from protected pyrrole **60**. (a) NBS, H_2_O, acetone, then (b) Et_3_N; (c) *N*-methyl aniline, Et_3_N, EtOAc, then (d) BF_3_, EtOAc; (e) POCl_3_, DMF; (f) Me_2_NH·HCl, DMF, then (g) aq. Na_2_CO_3_.

The following year, a novel methodology for the synthesis of cryptolepine was designed by Abe et al. (Scheme 12) [35]. One-pot functionalization of the double bond on tosyl indole **60** provided ammonium salt **61** in a very good yield. Substitution with *N*-methyl aniline and further dehydration resulted in the 3-substituted intermediate **62**. Vilsmeier–Haack formylation at indole C(2) afforded **63**, which underwent *N*-deprotection and ring closure to yield natural cryptolepine, with a 34% overall yield.

**Scheme 13:**
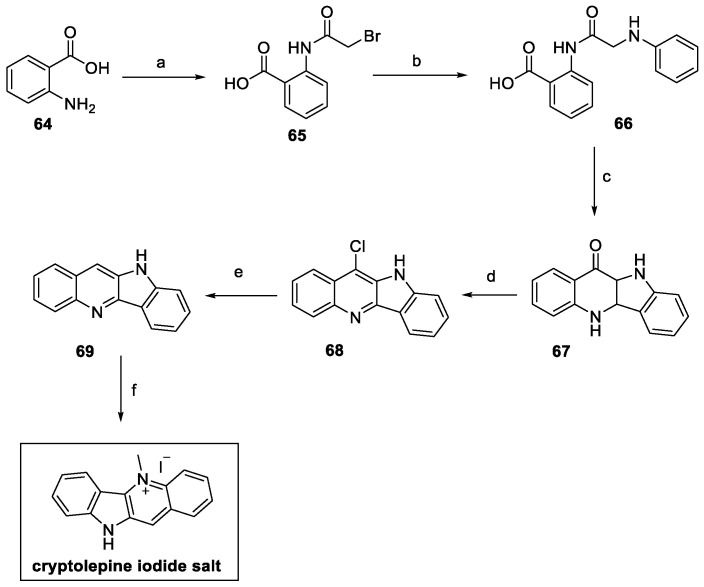
Synthesis of cryptolepine, starting from anthranilic acid **64**: (a) bromoacetyl bromide, 1,4-dioxane/DMF 1:1; (b) aniline, DMF; (c) PPA; (d) POCl_3_; (e) H_2_, Pd/C, AcOH, NaOAc, 60 psi; (f) MeI, DMF.

Following a different approach, cryptolepine was synthesized by Mudududdla et al., starting from commercially available anthranilic acid **64**, as depicted in Scheme 13 [36]. Bromoacetylation and alkylation with aniline led to benzoic acid **66**. Friedel–Crafts reaction and subsequent dehydration–cyclization catalyzed by polyphosphoric acid (PPA) provided access to indolo[*3,2-b*]quinolin-11-one **67**. The POCl_3_-mediated chlorination yieldied the quinoline skeleton on **68**, hydrogenated at high pressure, in the presence of a catalytic amount of Pd, to afford compound **69**. Final *N*-methylation with methyl iodide yielded cryptolepine iodide salt, with an overall yield of 16%, starting from anthranilic acid.

#### 2.4.2. Neocryptolepine

**Scheme 14:**
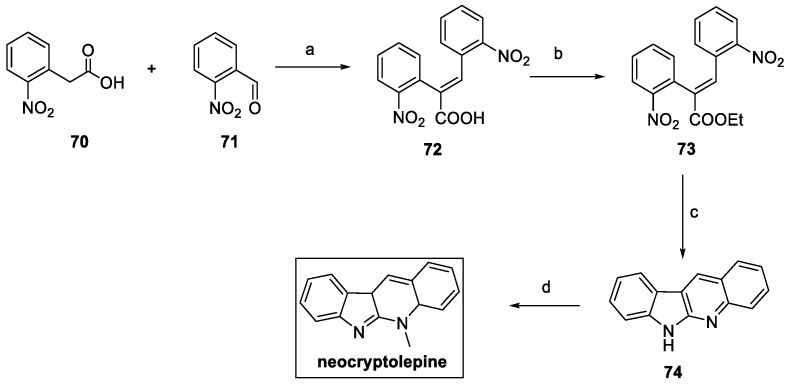
Synthesis of neocryptolepine. (a) Ac_2_O, Et_3_N, reflux; (b) EtOH, H_2_SO_4_, 24 h; (c) Fe/HCl, AcOH:EtOH:H_2_O; (d) Me_2_SO_4_, DMF, μW, or Me_2_SO_4_, MeCN.

Neocryptolepine was synthesized following a domino strategy, starting from *o*-nitrophenylacetic acid **70**. (Scheme 14) [31]. Perkin reaction with *o*-nitrobenzaldehyde **71** yielded the corresponding α,β-unsaturated acid **72**, further converted to ethyl ester **73**, in high yield. Iron powder in the presence of HCl allowed for the nitro reduction and ring closure via a domino reaction sequence, resulting in indoloquinoline **74**, a naturally occurring alkaloid also known as norcryptotackieine. Methylation of the quinoline nitrogen using dimethyl sulfate afforded neocryptolepine in around a 40% overall yield in four steps.

**Scheme 15:**
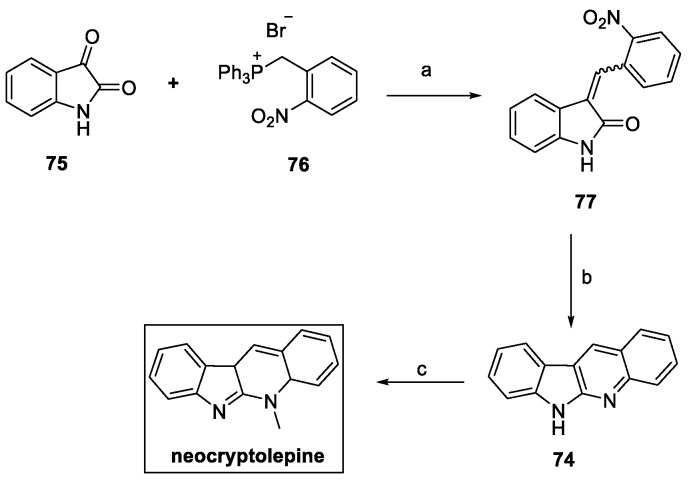
Optimized synthesis of neocryptolepine. (a) Et_3_N, CHCl_3_; (b) Fe/HCl, AcOH; (c) MeI, THF.

With the purpose of improving the overall yield of the pathway, an alternative method for the preparation of neocryptolepine was reported by the same authors (Scheme 15). In this case, Wittig olefination on isatin **75** with the phosphonium salt **76** yielded a 2:1 ratio of *E*/*Z* isomers (**77**). Reduction of the resulting olefin with iron powder in an acidic environment provided the fused tetracycle **74**, which was finally methylated to afford neocryptolepine with a 68% overall yield in just three steps [31].

**Scheme 16:**
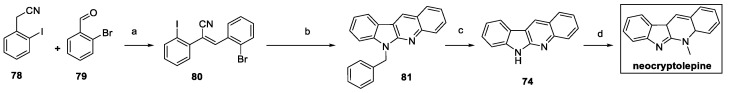
Synthesis of neocryptolepine, starting from cyano substrate **78**. (a) K_2_CO_3_, MeOH; (b) benzyl amine, Pd_2_(dba)_3_, xantphos, *t*-BuONa, toluene; (c) anhydrous AlCl_3_, benzene; (d) MeI, aq. KOH, THF [37].

In 2023, neocryptolepine was synthesized by exploiting different substrates using a similar approach (Scheme 16) [37]. Condensation between commercially available 2-iodobenzyl cyanide **78** and 2-bromobenzaldehyde **79** delivered (*E*)-stilbene intermediate **80**. Sequential Pd-catalyzed intermolecular and intramolecular double Buchwald−Hartwig C−N coupling with benzyl amine yielded the desired *N*^6^-benzylated norcryptotackieine **81**. AlCl_3_-catalyzed debenzylation resulted in norcryptotackieine **74**, which was finally methylated to neocryptolepine.

**Scheme 17:**
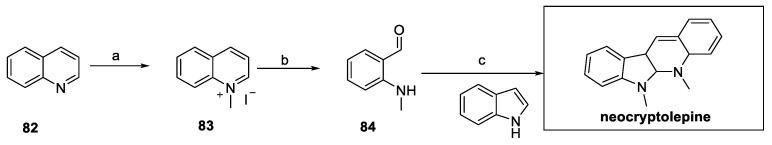
Synthesis of neocryptolepine, starting from substituted indole **82**. (a) *i*PrOH, MeI; (b) KOH, H_2_O_2_; (c) indole, *p*-TSOH, EtOH.

Neocryptolepine and its derivatives were synthetized by Zhu’s group and screened as fungicides against *R. solani*, *B. cinerea*, *F. graminearum*, *M. melonis*, *S. sclerotiorum*, and *M. oryzae* [38]. In particular, quinoline **82** was converted to the quinolonium salt **83** and underwent oxidative cleavage to access amino benzaldehyde **84** (Scheme 17). In this case, the tetrahydroquinoline moiety was obtained by treatment with indole in the presence of catalytic amounts of *p*-toluenesulfonic acid, thus affording the desired natural product.

#### 2.4.3. Isocryptolepine

**Scheme 18:**
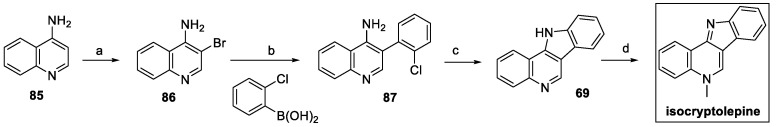
Synthesis of isocryptolepine, starting from 4-amino quinoline **85**. (a) Br_2_, AcOH; (b) Pd(PPh_3_)_4_, K_2_CO_3_, toluene:EtOH:H_2_O 3:2:1; (c) *t*-BuOK, DMSO; (d) MeI, toluene.

Isolation of isocryptolepine from the natural source (*Cryptolepis sanguinolenta*) and its chemical synthesis were thoroughly reported by Thobokholt et al. in their review in 2020 [39]. However, in 2021 a new, more efficient method was proposed using modifications of commercially available 4-aminoquinoline **85** (Scheme 18) [40]. Bromination at C(3) was followed by Suzuki–Miyaura coupling with 2-chlorophenylboronic acid to yield **87** in a very high yield. Intramolecular nucleophilic aromatic substitution provided indoloquinoline **69**, which was finally methylated to isocryptolepine, with a 61% overall yield in four steps.

## 3. Indole-Containing Alkaloids

### 3.1. β-Carbolines: Harmine, Norharmane, Harmaline, Harmol, Harmane, and Harmalol (from Plants)

**Figure 6:**
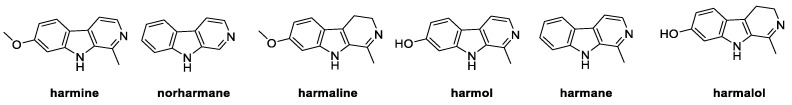
Chemical structure of natural β-carbolines.

β-carbolines belong to a class of natural alkaloids widely distributed in plants and food, as well as in marine microorganisms, insects, and mammalians. Isolated from *Peganum harmala* for the first time in 1841, these heterocyclic compounds are characterized by a 9*H*-pyrido[*3,4-b*]indole core [41], endowed with antifungal activity. Six natural carbolines, namely harmine, norharmane, harmaline, harmol, harmane, and harmalol, were studied for their growth inhibition effect on strains of *P. digitatum*, *F. oxysporum*, and *B. cinerea*, which are mainly caused by cellular membrane damage and ROS accumulation (Figure 6) [41].

**Scheme 19:**
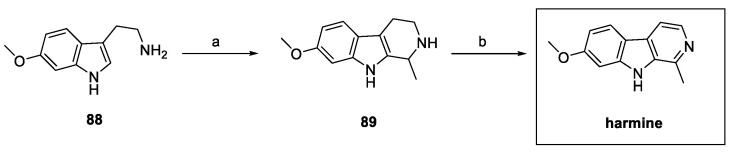
Classical synthesis of harmine via Pictet–Spengler reaction: (a) 0.1 N HCl, acetaldehyde; (b) 5% Pd/C, dry toluene.

In recent years, different authors have reported the synthesis of harmine, commonly obtained by a Pictet–Spengler reaction between previously prepared 6-methoxy tryptamine **88** (obtained in four steps) [42] and acetaldehyde, followed by dehydrogenation on a Pd/C catalyst (Scheme 19) [43].

**Scheme 20:**
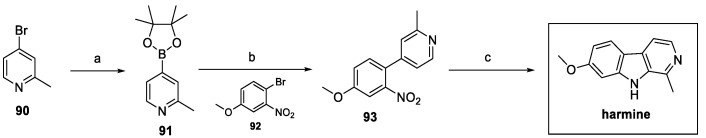
Synthesis of harmine via Suzuki reaction: (a) bis(pinacolato)diboron, Pd(dppf)Cl_2_, KOAc, dry 1,4-dioxane; (b) Pd(dppf)Cl_2_, Cs_2_CO_3_, 1,4-dioxane/H_2_O; (c) PPh_3_, *o*-DCB.

In order to design a shorter synthesis of harmine, W. Liu et al. proposed a new three-step route, as shown in Scheme 20. Starting from 4-bromo-2-methylpyridine **90**, borylation and Suzuki coupling with the suitable bromide **92** afforded aryl pyridine **93**, which then underwent Cadogan cyclization to obtain the desired carboline harmine [44].

**Scheme 21:**
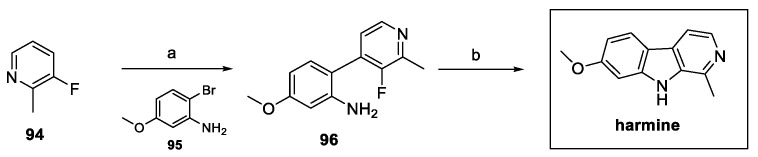
Synthesis of harmine via Negishi coupling: (a) i. LDA, THF; ii. ZnCl*_2_*; iii. Pd XPhos G3, THF; (b) NaHMDS, THF.

Several natural β-carbolines, including harmine, were synthesized in two steps, following the approach proposed by Sathiyalingama et al. (Scheme 21). Direct harmine precursor **96** was obtained via tandem reaction (lithiation, transmetalation, Negishi coupling), starting from fluoropyridine **94**. Final intramolecular nucleophilic aromatic substitution resulted in the desired natural compound [45].

### 3.2. Pityriacitrin and Pityriacitrin B (from Bacteria and Marine Environment)

**Figure 7:**
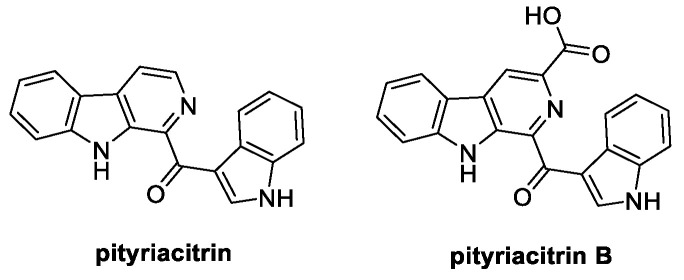
Chemical structures of pityriacitrin and pityriacitrin B.

Characterized by a β-carboline core structure bearing an acyl portion at C(1), pityriacitrin was first extracted and isolated from the marine bacterium *Paracoccus* sp. in 1999 (Figure 7) [46]. Instead, its carboxylated analogue pityriacitrin B naturally occurs in the human pathogenic yeast *Malassezia furfur*; moreover, it is known as a secondary metabolite obtained from the marine fungus *Dichotomomyces cejpii* and from Chinese *Burkholderia* sp. NBF227.

Antimalarial, antitumor, antioxidant, PLA2 regulation, and UV protection are among the biological activities reported for pityriacitrin, even though its application as a crop protector has only recently been described. Indeed, pityriacitrin significantly inhibits the growth rate of *R. cerealis* and *S. sclerotiorum* [47].

**Scheme 22:**
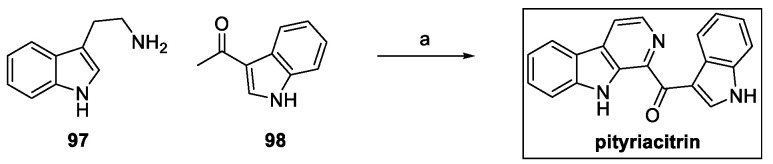
Synthesis of pityriacitrin: (a) I_2_, DMSO.

So far, five total syntheses have been reported in the literature, starting from the valuable work regarding the one-pot approach by Zhu et al. using tryptamine **97** and the carbonyl indole **98** under oxidative reaction conditions (Scheme 22) [48].

**Scheme 23:**
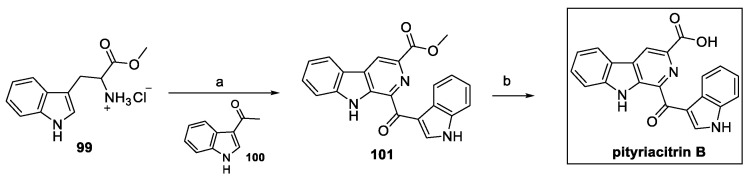
Synthesis of carboline pityriacitrin B. (a) I_2_, DMSO; (b) NaOH, MeOH, H_2_O.

Starting from tryptophan methyl ester **99** allowed for the introduction of a carboxylic moiety so that heterocyclization with acetyl indole **100** and further hydrolysis could afford pityriacitrin B (Scheme 23) [49].

### 3.3. Meridianin (from Marine Environment)

**Figure 8:**
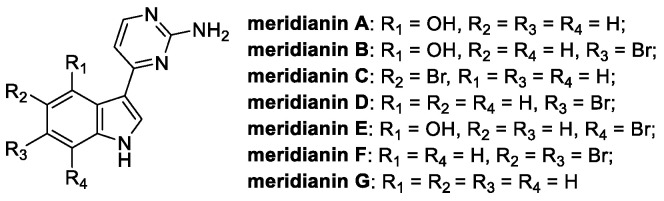
Chemical structure of meridianins A-G [51].

Meridianins are alkaloids isolated from marine organisms like the Antarctic tunicates *Aplidium meridianum*, *falklandicum,* and *Synoicum* sp. [50]. Starting in 1998, a total of eight meridianins have been collected and chemically characterized, with the core structure typically consisting of a brominated or hydroxylated indole, substituted at C(3) with a 2-aminopyrimidine moiety (Figure 8) [50].

Meridianins C, D, and G are endowed with a plethora of biological activities (anticancer, protein kinase inhibitory, antimalarial, antituberculosis, and anti-Alzheimer), including the fungicidal type [52]. Specifically, meridianin C has been reported as the most active, showing a broad-spectrum antifungal activity against several phytopathogenic fungal strains [51].

**Scheme 24:**
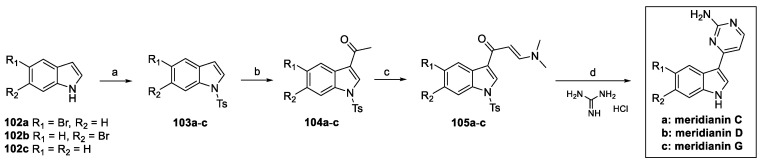
(a) TsCl, NaOH, H_2_O, *n*-Bu_4_NHSO_4_, toluene; (b) Ac_2_O, AlCl_3_, DCM; (c) DMF-DMA; (d) K_2_CO_3_, CH_3_OCH_2_CH_2_OH.

To date, several papers concerning the total synthesis of meridianins have been published [53,54,55]. Among them, the strategy reported by Molina, based on the Bredereck protocol, will hereafter be described [54]. Meridianin C, D, and G were synthetized, starting from the substituted indoles **102a**–**c** (Scheme 24). Nitrogen tosylation and C(3)-acylation afforded **104a**–**c**, which were further functionalized to obtain enaminones **105a**–**c**. Bredereck cyclization and *N*-deprotection finally afforded the desired natural products [51].

## 4. Miscellanea

### 4.1. Phenylpirrole: Pyrrolnitrin (from Bacteria)

**Figure 9:**
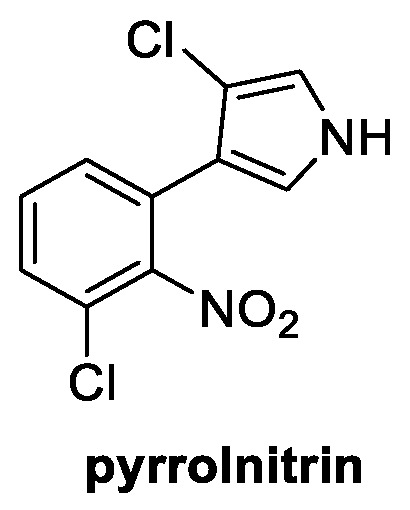
Chemical structure of pyrrolnitrin.

Pyrrolnitrin is an interesting natural compound well-known for its antifungal properties. Some of its derivatives are, in fact, already widely used in agriculture. It was firstly isolated in 1964 from *Pseudomonas pyrrocinia* and was later also identified in several other proteobacterial strains [56]. It is structurally characterized by a chlorinated pyrrole ring linked to a 2-nitro-3-cloro phenyl group (Figure 9). Pyrrolnitrin is known to reach the intracellular environment of the fungus via passive diffusion, targeting the mitochondrial electron transport chain. The increased oxidative stress and proliferation of oxygen free radicals are responsible for the damage occurring from the spore germination stage (inhibition of conidial germination at 0.4 µg/mL concentration) to the mycelia growth phase [57]. It is considered a broad-spectrum fungicide, with its strongest activity reported against *Alternaria* sp., *B. cinerea*, *P. aphanidermatum*, *P. ultimun*, *R. solani*, *Rhizopus* sp., *A. niger*, *F. oxysporum*, *P. expansum*, and *S. rolfsii* [58].

**Scheme 25:**
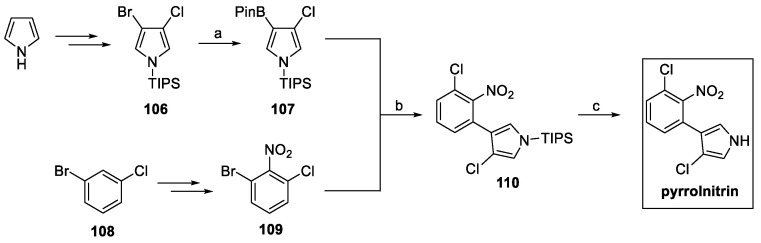
Synthesis of pyrrolnitrin. (a) HBPin, Et_3_N; (b) Pd(OAc)_2_:SPhos (1:2), K_3_PO_4_, *n*-BuOH:H_2_O; (c) TBAF, THF.

Pyrrolnitrin total synthesis was first approached by Nakano et al. in 1966 [59]. However, newer and more versatile approaches have been developed over the years, including the overexpression of its gene cluster in *E. coli* [60]. Morrison et al. reported a convenient synthetic pathway, starting from protected pyrrole (Scheme 25) [60].

Following the protocol previously described by Muchowski et al. [61], brominated pyrrole **106** was converted to boronate ester **107**, ready to undergo Suzuki coupling with the suitable aromatic substrate, in this case, nitro compound **109,** obtained starting from aryl halide **108**. Final TBAF-mediated deprotection afforded pyrrolnitrin [60].

### 4.2. Triazoles: Penipanoid A (from Bacteria)

**Figure 10:**
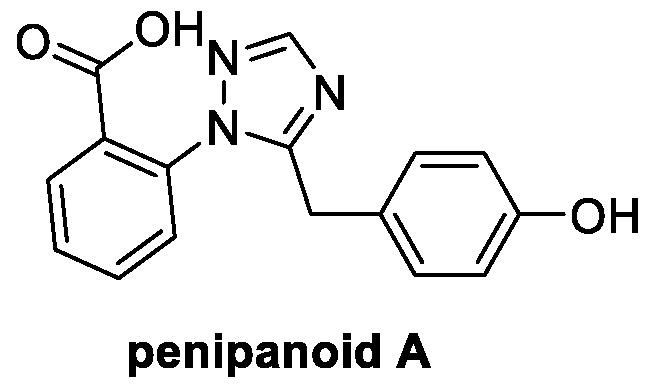
Chemical structure of penipanoid A.

Penipanoid A is a natural product isolated from the marine fungus *Penicillium paneum* SD-44, found in a deep-sea sediment of the South China Sea [62]. Its main structural feature is a 1,2,4-triazole ring, widely present in a huge number of fungicides, such as fluconazole and voriconazole, bearing a benzoic acid and a phenol ring (Figure 10) [63]. To date, cytotoxic and antimicrobial activities have been reported, and further investigations are needed to assess its fungicidal potential.

**Scheme 26:**
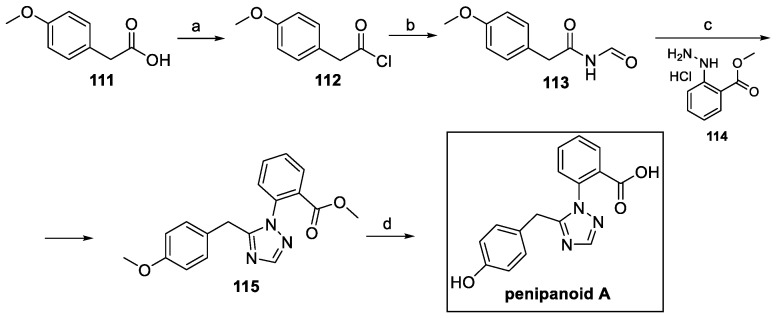
Synthesis of penipanoid A, starting from 4-methoxy phenylacetic acid **111**. (a) (COCl)_2_; (b) formamide, Py, acetone; (c) **114**, AcOH; (d) BBr_3_, DCM.

The total synthesis of this compound was reported in 2022 [63]. At first, commercially available phenylacetic acid **111** was activated and reacted with formamide to yield key intermediate **113** (Scheme 26). The triazole moiety of **115** was achieved by cyclization with phenyl hydrazine **114**. Finally, ester hydrolysis, followed by methyl ether deprotection, allowed for the obtainment of penipanoid A [63].

**Scheme 27:**
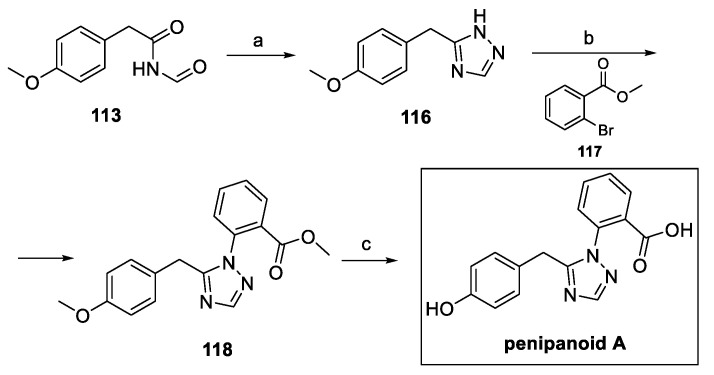
Synthesis of penipanoid A, starting from intermediate **113**. (a) NH_2_-NH_2_, AcOH; (b) **117**, CuI, Cs_2_CO_3_, L-proline, DMF; (c) BBr_3_, DCM.

The same authors described further improvements in the synthetic pathway, starting from intermediate **113**. Construction of the triazole ring was afforded in the presence of hydrazine. Ullmann reaction with 2-bromomethyl benzoate **117** yielded the 1,5-disubstituted desired moiety **118**, which was completely demethylated to obtain pure penipanoid A, with a 60% overall yield, while shortening the synthetic pathway by one step (Scheme 27).

**Scheme 28:**
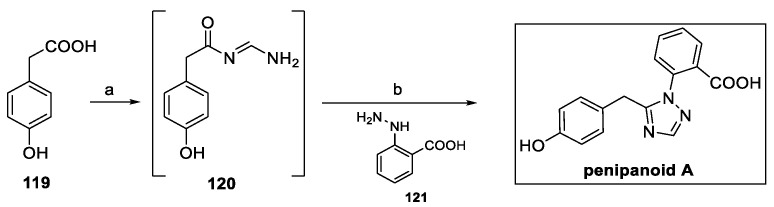
Protection-free synthesis of penipanoid A: (a) formamidine hydrochloride, HATU, DIPEA, DMF; (b) **121**, AcOH.

Recently a new one-pot strategy for the preparation of penipanoid A was designed to allow for a more accessible preparation of new analogs and to provide the opportunity to investigate their structure–activity relationships as antifungal agents [64]. Exploiting such an approach, 4-hydroxyphenyl acetic acid **119** was converted to intermediate **120** in the presence of formamide (Scheme 28). The addition of 2-hydrazinylbenzoic acid hydrochloride **121** finally afforded the product in a 74% yield, avoiding any protection–deprotection sequence.

### 4.3. Naphthimidazoles: Kealiinines (from Marine Environment)

**Figure 11:**
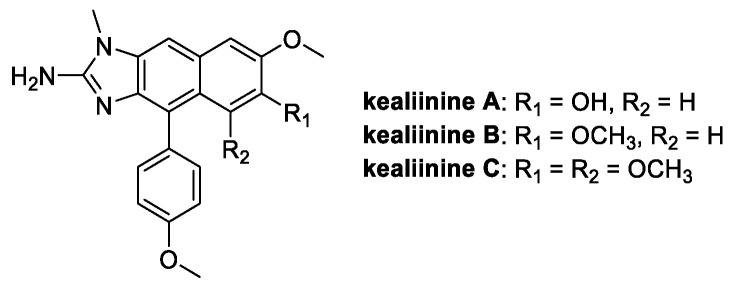
Chemical structure of kealiinine A–C.

Kealiinines A-C (Figure 11) are alkaloids isolated from the Indonesian sponge *Leucetta chagosensis* and studied since 2013 for their antitumor properties [65]. Among these, kealiinine B, with its 2-aminonaphthimidazole core, is endowed with the highest antimicrobial activity in the field of crop protection, performing better than ribavirin on tobacco mosaic virus, and suppressing the growth rate of *P. capisci* (68%), *P. piricola* (63%), and *R. solani* (61%) [66].

**Scheme 29:**
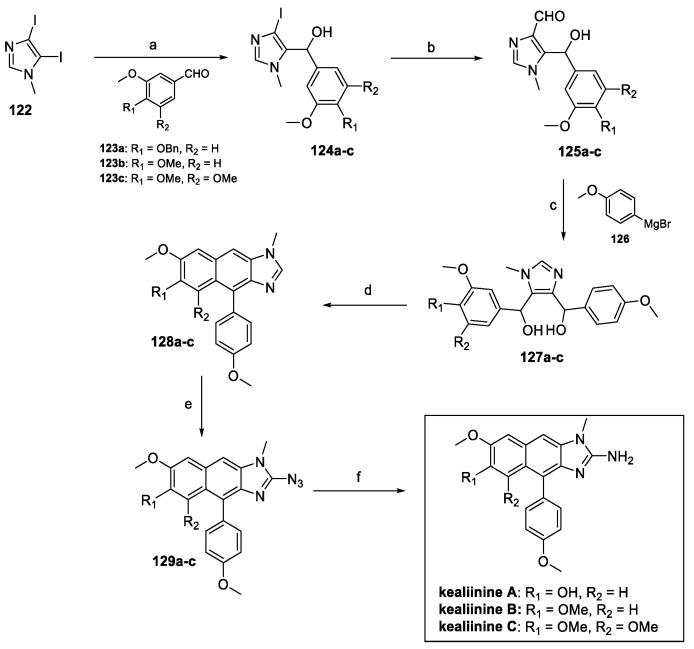
(a) EtMgBr/THF; (b) *N*-methyl formanilide, EtMgBr/THF; (c) **126**, THF; (d) HCl, DCM; (e) i *n*-BuLi, THF; ii TsN_3_; (f) Pd-C, H_2_, MeOH/THF.

The total synthesis of kealiinine B has been reported by different groups, mainly exploiting the method developed by Das et al [67]. 4,5-diiiodo-1-methyl-1*H*-imidazole **122** reacted with differently substituted benzaldehydes to afford aryl alcohol **123a**–**c** (Scheme 29). Formylation at C(4) of the imidazole core afforded hydroxy aldehyde **125a**–**c**, ready to undergo nucleophilic addition via Grignard reagent **126**. Ring closure and an additional dehydration–aromatization step led to intermediate **128a**–**c**. Lithiation and imidazole azidolysis at C(2) resulted in compound **129a**–**c**, giving access, upon Pd catalyzed hydrogenation, to kealiinine A–C.

### 4.4. Aminoimidazoles: Naamines and Naamidines (from Marine Environment)

**Figure 12:**
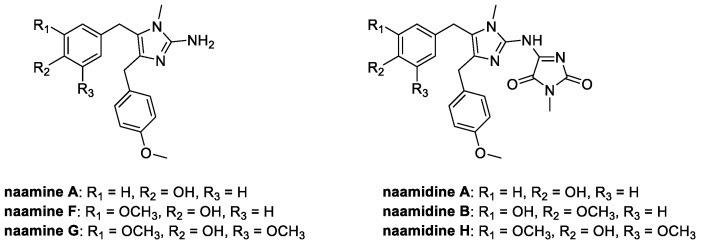
Chemical structure of naamines and naamidines.

Together with kealiinines, *Leucetta chagosensis* is also the natural source of a series of alkaloids having the 2-amino imidazole ring in common (Figure 12). These molecules, called naamines and naamidines, are known for their interesting biological activities, ranging from antitumoral to nitric oxide synthase inhibition; moreover, they are characterized by the ability to prevent the spread of plant diseases [68].

**Scheme 30:**
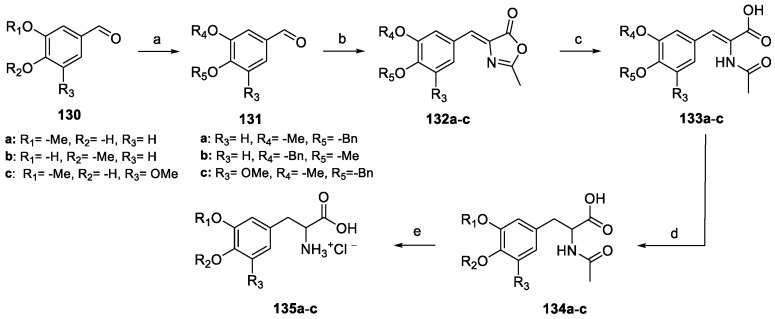
Synthesis of amino acids **135a**–**c**. (a) BnBr, MeOH, K_2_CO_3_; (b) *N*-acetylglycine, AcONa, Ac_2_O; (c) (i) aqueous NaOH; (ii) aqueous HCl; (d) Pd/C, H_2_; (e) HCl conc.

**Scheme 31:**
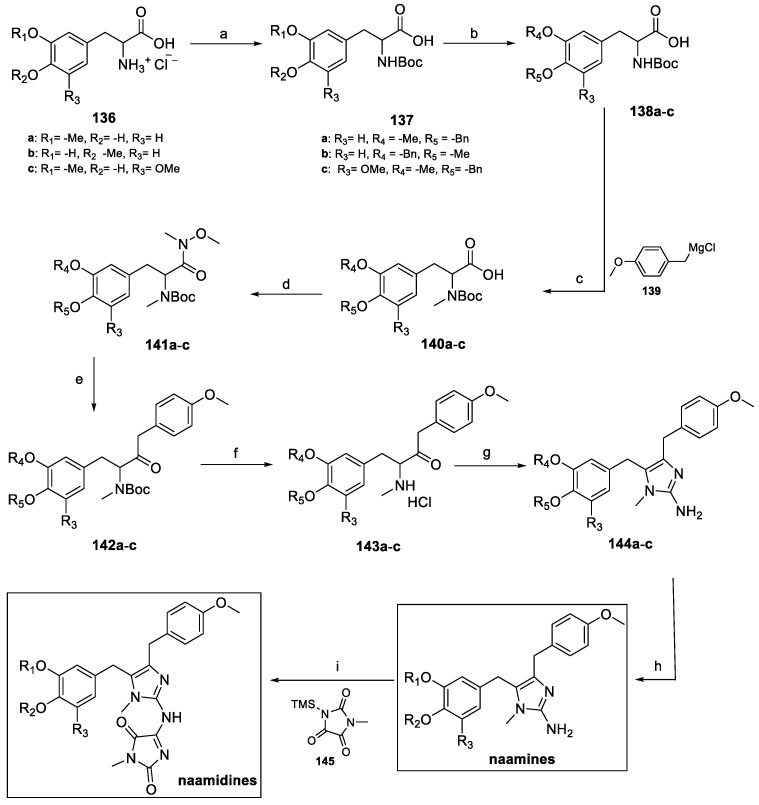
Synthetic approaches to naamines and naamidines. (a) Boc_2_O, NEt_3_, 1,4-dioxane, H_2_O; (b) BnBr, K_2_CO_3_, MeOH; (c) MeI, NaH, THF; (d) *N*,*O*-dimethylhydroxylamine hydrochloride, DIPEA, HOBt, EDCI, DCM; (e) **139**, THF, Et_2_O; (f) HCl, H_2_O; (g) NH_2_CN, H_2_O, EtOH; (h) H_2_, Pd/C, MeOH, AcOH; (i) toluene.

Naamine A and naamidine A were first synthetized in 2000 by Ohta et al [69], while naamines B [70], C, E, G [71], and naamidines G–H [72,73] were prepared by imidazole metalation or alkyne amination (Scheme 30 and Scheme 31).

Once protected as benzyl ethers, benzaldehyde **131a**–**c** reacted with *N*-acetyl glycine to yield oxazolones **132a**–**c**. Alkaline hydrolysis and further catalytic hydrogenation yielded carboxylic acids **134a**–**c**, which were finally deacetylated under acidic conditions to amino acids **135a**–**c**.

**Scheme 32:**
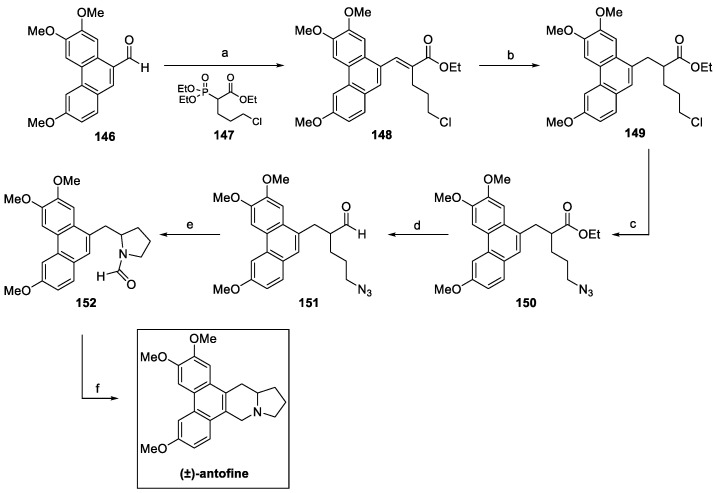
Synthesis of (±)-antofine. (a) NaH, THF; (b) H_2_, Pd/C, EtOAc, then (c) NaN_3_, TBAI, DMF; (d) DIBAL-H, DCM; (e) TFA, DCM, then (f) HCHO, HCl EtOH.

BOC protection of **137a**–**c** was required to methylate the imidazole NH group, while Weinreb amides **141a**–**c** were obtained by treatment with *N*,*O*-dimethylhydroxylamine hydrochloride (Scheme 32). Nucleophilic substitution with the Grignard reagent **139**, followed by BOC deprotection afforded the methylamino hydrochloride salts **143a**–**c**. Treatment with cyanamide allowed for the formation of the aminoimidazole moiety (compound **144a**–**c**), which was finally debenzylated to yield naamines. Further functionalization with methyl parabanic acid **145** afforded naamidines.

### 4.5. Indolizidines: Antofine (from Plants)

**Figure 13:**
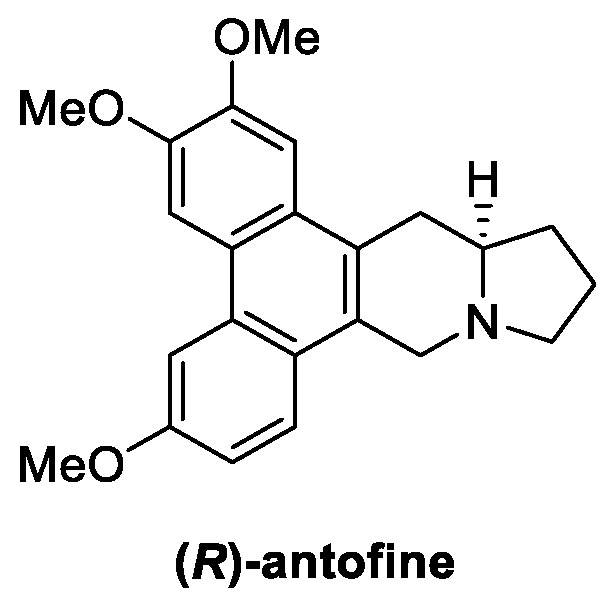
Chemical structure of (*R*)-antofine.

Antofine (Figure 13) is a bioactive alkaloid extracted from *Cynanchum atratum* (Bunge), belonging to the family *Asclepiadaceae* and widely used in Chinese traditional medicine for its antipyretic, antioxidant, anti-inflammatory, and antiviral properties [74,75,76,77]. In the last decade, antifungal potential of antofine was investigated by Wang et al., revealing significant activity, with inhibition rates up to 100%, against *Alternaria solani*, *Cercospora arachidicola*, *Physalospora piricola*, and *Cladosporium cucumerium* at 50 μg/mL [78]. Furthermore, a dose-dependent growth inhibition of *Penicillium italicum* (MIC = 1.56 µg/mL) was recently reported as result of plasma membrane perturbation, the reduction of ergosterol biosynthesis, and the disruption of the Krebs cycle in the mycelia [74,79].

In 2014, M. Yi et al. reported an efficient preparation of (±)-antofine in six steps from phenanthryl aldehyde **146**, with an overall yield of 35%, as shown in Scheme 32 [80]. The Horner–Wadsworth–Emmons reaction with phosphonate **147** yielded the α,β-unsaturated ester **148**, further hydrogenated and converted to terminal azide **150**. Reduction of the ester to aldehyde **151** was followed by a TFA-promoted intramolecular Schmidt reaction to yield the desired formamide **152**, which underwent intramolecular ring closure in the presence of formaldehyde, according to the Pictet–Spengler protocol, to furnish (±)-antofine.

**Scheme 33:**
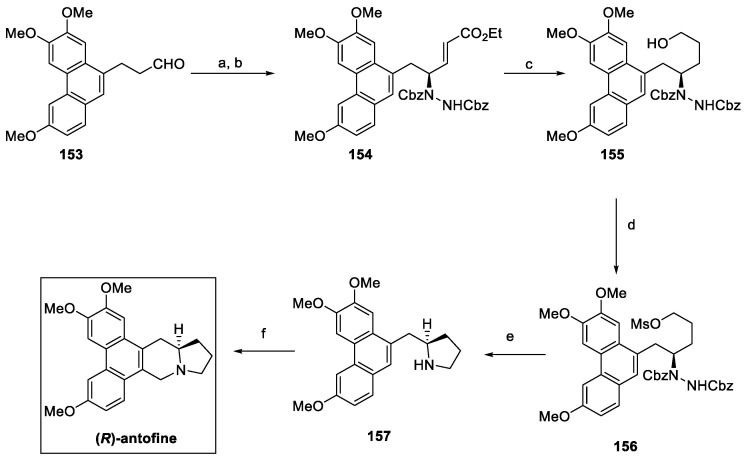
Synthesis of enantiopure (*R*)-antofine. (a) CbzN=NCbz, D-Proline, CH_3_CN, DCM, then (b) Ph_3_P=CHCOOEt, 94% *ee*; (c) LiBH_4_, THF; (d) MsCl, Et_3_N, DMAP, DCM; (e) H_2_, Raney Ni, MeOH; (f) HCHO, HCl, EtOH.

In 2018, an enantioselective approach to (*R*)-antofine was reported, starting from aldehyde **153** (Scheme 33) [81]. Asymmetric α-hydrazination with dibenzyl azodicarboxylate (DBAD) in the presence of D-proline was followed by reaction with the suitable phosphorane to yield the α,β-unsaturated ester **154**, with a 94% enantiomeric excess. Reduction of **154** with LiBH_4_ resulted in the primary alcohol **155**, which was further converted to mesylate **156**. Cbz deprotection by hydrogenolysis over a Raney Ni catalyst favored the intramolecular substitution to the enantiomerically pure pyrrolidine derivative **157**. The Pictet–Spengler reaction finally yielded (*R*)-antofine, with an overall yield of 58% in five steps.

### 4.6. Pyrimidines: Essramycin (from Marine Environment)

**Figure 14:**
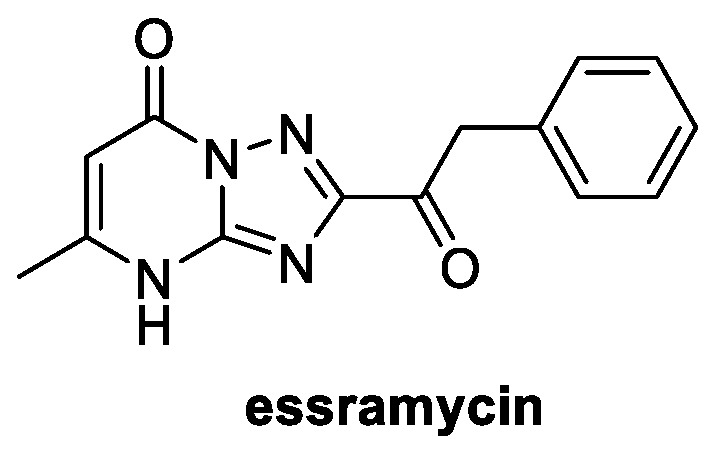
Structure of essramycin.

Essramycin was isolated from the fermentation broth of *Streptomyces* sp. Merv8102, a marine organism found in sediments from the Mediterranean Sea off the Egyptian coast [82]. Its structure is quite unique for a natural product, since it is claimed to be the first one assigned with a 1,2,4-triazolo[1,5-α]pyrimidine core (Figure 14) [83].

Pyrimidoquinolines are endowed with important biological properties such as anticancer, phosphodiesterase inhibiting, antitubercular, antiepileptic, antimalarial, and antibacterial activities [84]. As for essramycin, promising results were observed against *E. coli*, *P. aeruginosa*, *B. subtilis*, *S. aureus*, and *M. luteus*; however, its structural similarity with ribavirin suggested its possible application as an antiviral compound for crop protection against tobacco mosaic virus [85]. Recently, essramycin was screened as a fungicide against a set of phytopathogenic fungi, showing inhibition rates between 45 and 60% against *B. cinerea*, *P. infestans*, and *R. cerealis* at a 50 µg/mL concentration [86].

**Scheme 34:**
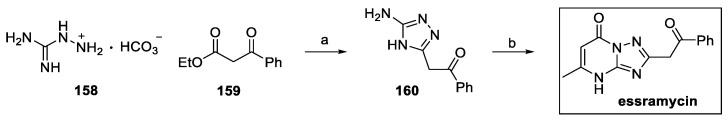
Synthesis of essramycin: (a) *n*-butanol; (b) ethyl acetoacetate, glacial AcOH.

To date, the only protocol reported for the preparation of essramycin consists of a two-step synthesis, starting from aminoguanidine bicarbonate **158** and ethyl benzoylacetate **159** (Scheme 34) [83]. Cyclization to 1,2,4-triazole intermediate **160** was followed by condensation with ethyl acetoacetate, affording the pyrimidoquinoline core of essramycin in a 35% yield.

### 4.7. Phenazines: Phenazine-1-Carboylic Acid, PCA (from Bacteria)

**Figure 15:**
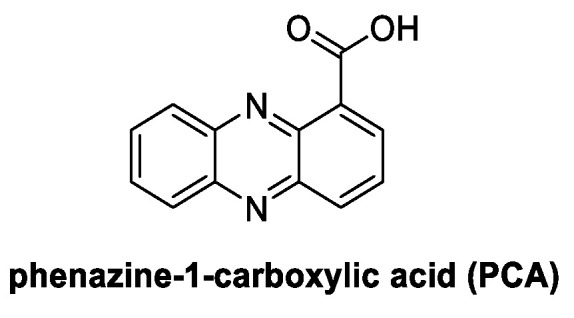
Chemical structure of phenazine-1-carboxylic acid (PCA).

Phenazine-1-carboxylic acid (PCA) is a relevant bacterium-derived natural compound which is well known for its antifungal activity and applications in crop protection (Figure 15) [87]. Isolated as a metabolite from bacteria of the genera *Pseudomonas* and *Streptomyces*, it is currently registered in China as Shenqinbactin. It acts as a biofungicide characterized by high efficacy (related to the disruption of redox homeostasis in fungal cells) [88] and associated with low human, animal, and environmental toxicity [88].

**Scheme 35:**
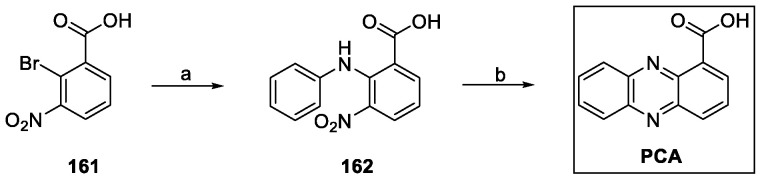
Synthesis of phenazine-1-carboxylic acid (PCA): (a) aniline, CuCl_2_, copper powder, *N*-ethyl morpholine, 2,3-butanediol; (b) NaBH_4_, EtONa, EtOH.

Xiong et al. described the synthesis of PCA, starting with an Ullmann reaction between aniline and the bromo benzoate **161** to afford **162**. Reduction of the nitro group readily produced ring closure, with subsequent formation of phenazine-1-carboxamide (Scheme 35) [89,90].

## 5. Antifungal Activity

All the described natural compounds were assayed in terms of antifungal activity on several fungal strains. The data discussed in the previous paragraphs have been summarized in Table 1, indicating the phytopathogen and the tested alkaloid, together with the percentage of mycelia growth inhibition, the minimum inhibitory concentration (MIC), or the half maximal effective concentration (EC_50_).

## 6. Conclusions

Alkaloids represent a promising class of natural antifungal agents for crop protection. With the increasing demand for eco-friendly agricultural practices, research into the development and commercialization of alkaloid-based fungicides could provide a valuable tool for managing fungal diseases in crops.

Synthesizing alkaloids for agricultural use can be beneficial for several reasons, including the overcoming of supply and extraction limitations and improving cost-effectiveness and scalability.

Over the last ten years, numerous methods have been exploited for the synthesis of polycyclic nitrogen-containing moieties, usually with good overall yields and a reasonable number of steps. Moreover, remarkable advances have been reported, especially for the synthesis of enantiomerically pure compounds, exploiting chiral species in catalytic amounts and cascade/domino reactions. Such efforts allowed for the efficient customization and optimization of chemical structures, which is both advantageous and convenient for the preparation of new nature-derived chemotypes, potentially useful as valuable tools in advancing agricultural practices while safeguarding ecological balance. However, as for the synthesis of other natural compounds, the chemical complexity of alkaloids (e.g., multiple stereogenic centers, fused rings), in most cases, still requires multistep processes affecting the overall yield of the synthetic approach. Moreover, the catalysts, reagents, and reaction conditions required could be cost-intensive and must be followed by purification and isolations steps, which are not always scalable for an industrial process.

The fascinating complexity of natural biosynthesis is still difficult to reproduce in a laboratory, but since natural compounds represent the most replenished arsenal against pathogens, researchers from all over the world will continue researching innovative and revolutionary paths to mimic what nature has mastered over millennia.

## Figures and Tables

**Table 1:** Antifungal activity of all the synthesized compounds reported. All the compounds were evaluated using the mycelium growth rate method (procedures in the corresponding references), except for PCA, which was evaluated via the hyphae growth velocity assay.

Phytopathogens	Natural Alkaloids	Mycelia Growth Inhibition	MIC	EC_50_	Ref.
*F. graminearum*	Quinine	46% at 100 μg/mL			[15]
	Neocryptolepine			16.31 μg/mL	[38]
	Meridianin C, D, G	58% at 50 μg/mL			[51]
	Naamines and naamidines	36% at 50 μg/mL			[68]
	Kealiinine A, B, C	24% at 50 mg/kg			[66]
*P. zeae*	Quinine	30% at 100 μg/mL			[15]
	Isocryptolepine	35% at 100 μg/mL			[34]
*R. solani*	Quinine	30% at 100 μg/mL			[15]
	Sanguinarine	77% at 50 μg/mL		11.6 μg/mL	[91]
	Pyrrolnitrin		1 μg/mL		[58]
	Neocryptolepine			9 μg/mL	[38]
	Isocryptolepine	6% at 100 μg/mL			[34]
	Meridianin C, D, G	72% at 50 μg/mL			[51]
	Naamines and naamidines	47% at 50 μg/mL			[68]
	Kealiinine A, B, C	61% at 50 mg/kg			[66]
	PCA			25.66 ± 1.75 μg/mL	[88]
*M. melonis*	Quinine	30% at 100 μg/mL			[15]
	Neocryptolepine			1.16 μg/mL	[38]
*B. cinerea*	Luotonin A			0.50 mM	[24]
	Cryptolepine			0.050 μg/mL	[32]
	Neocryptolepine			4.44 μg/mL	[38]
	Harmane		1 mM		[41]
	Harmine		0.5 mM		[41]
	Harmaline and harmalol	30% at 1 mM			[41]
	Pityriacitrin B	82% at 1 mg/mL			[47]
	Essramycin	46% at 50 mg/kg			[86]
	Pyrrolnitrin		5 μg/mL		[58]
	Meridianin C, D, G	68% at 50 μg/mL			[51]
	Naamines and naamidines	38% at 50 μg/mL			[68]
	Kealiinine A, B, C	11% at 50 mg/kg			[66]
*M. oryzae*	Quinine	30% at 100 μg/mL			[15]
	Luotonin A			0.50 mM	[24]
	Neocryptolepine			17.63 μg/mL	[38]
	Isocryptolepine	12% at 100 μg/mL			[34]
	Sanguinarine			6.96 μg/mL	[29]
*S. sclerotiorum*	Cryptolepine			5.507 μg/mL	[32]
	Neocryptolepine			17.65 μg/mL	[38]
	Pityriacitrin	52% at 50 μg/mL			[47]
	Meridianin C, D, G	70% at 50 μg/mL			[51]
	Naamines and naamidines	38% at 50 μg/mL			[68]
	Kealiinine A, B, C	35% at 50 mg/kg			[66]
*P. digitatum*	Antofine				[78]
	Harmane		1.56 μg/mL		[41]
	Harmine		0.5 mM		[41]
*R. cerealis*	Pityriacitrin	42% at 50 μg/mL	0.5 mM		[47]
	Essramycin	58% at 50 mg/kg			[86]
	Meridianin C, D, G	66% at 50 μg/mL			[51]
	Naamines and naamidines	70% at 50 μg/mL			[68]
	Kealiinine A, B, C	41% at 50 mg/kg			[66]
*P. infestans*	Essramycin	58% at 50 mg/kg			[86]
	Meridianin C, D, G	46% at 50 μg/mL			[51]
	Naamines and naamidines	25% at 50 μg/mL			[68]
	Kealiinine A, B, C	44% at 50 mg/kg			[66]
*Alternaria* sp.	Pyrrolnitrin		5 μg/mL		[58]
*A. solani*	Sanguinarine	68% at 50 μg/mL			[91]
	Meridianin C, D, G	62% at 50 μg/mL			[51]
	Naamines and naamidines	50% at 50 μg/mL			[68]
	Kealiinine A, B, C	13% at 50 mg/kg			[66]
*P. aphanidermatum*	Pyrrolnitrin		1 μg/mL		[58]
*P. ultimun*	Pyrrolnitrin		5 μg/mL		[58]
*Rhizopus* sp.	Pyrrolnitrin		1 μg/mL		[58]
*F. oxysporum*	Sanguinarine	15% at 50 μg/mL			[91]
	Pyrrolnitrin	85% at 20 μg/mL			[58]
*F. oxysporumf.* sp*. cucumeris*	Meridianin C, D, G	41% at 50 μg/mL			[51]
	Naamines and naamidines	20% at 50 μg/mL			[68]
	Kealiinine A, B, C	42% at 50 mg/kg			[66]
*P. expansum*	Pyrrolnitrin		20 μg/mL		[58]
*S. rolsfii*	Pyrrolnitrin		10 μg/mL		[58]
*P. piricola*	Meridianin C, D, G	88% at 50 μg/mL			[51]
	Naamines and naamidines	67% at 50 μg/mL			[68]
	Kealiinine A, B, C	63% at 50 mg/kg			[66]
*P. capsici*	Pityriacitrin B	78% at 1 mg/mL			[47]
	Kealiinine A, B, C	68% at 50 mg/kg			[66]
	Meridianin C, D, G	82% at 50 μg/mL			[51]
	Naamines and naamidines	17% at 50 μg/mL			[68]
*C. arachidicola Hori*	Sanguinarine	67% at 50 μg/mL			[91]
	Meridianin C, D, G	59% at 50 μg/mL			[51]
	Naamines and naamidines	47% at 50 μg/mL			[68]
	Kealiinine A, B, C	29% at 50 mg/kg			[66]
*B. maydis*	Meridianin C, D, G	36% at 50 μg/mL			[51]
	Naamines and naamidines	28% at 50 μg/mL			[68]
	Kealiinine A, B, C	22% at 50 mg/kg			[66]
*W. anthracnose*	Meridianin C, D, G	54% at 50 μg/mL			[51]
	Naamines and naamidines	33% at 50 μg/mL			[68]
	Kealiinine A, B, C	34% at 50 mg/kg			[66]
*F. moniliforme*	Meridianin C, D, G	24% at 50 μg/mL			[51]
	Naamines and naamidines	26% at 50 μg/mL			[68]
	Kealiinine A, B, C	19% at 50 mg/kg			[66]

## References

[1] FAO (2015). Achieving Zero Hunger: The Critical Role of Investments in Social Protection and Agriculture.

[2] Peng Y., Li S.J., Yan J., Tang Y., Cheng J.P., Gao A.J., Yao X., Ruan J.J., Xu B.L. (2021). Research Progress on Phytopathogenic Fungi and Their Role as Biocontrol Agents. Front. Microbiol..

[3] Zubrod J.P., Bundschuh M., Arts G., Brühl C.A., Imfeld G., Knäbel A., Payraudeau S., Rasmussen J.J., Rohr J., Scharmüller A. (2019). Fungicides: An Overlooked Pesticide Class?. Environ. Sci. Technol..

[4] Pérez-Pizá M.C., Sautua F.J., Szparaga A., Bohata A., Kocira S., Carmona M.A. (2024). New Tools for the Management of Fungal Pathogens in Extensive Cropping Systems for Friendly Environments. CRC Crit. Rev. Plant Sci..

[5] Tsalidis G.A. (2022). Human Health and Ecosystem Quality Benefits with Life Cycle Assessment Due to Fungicides Elimination in Agriculture. Sustainability.

[6] Yeshi K., Crayn D., Ritmejerytė E., Wangchuk P. (2022). Plant Secondary Metabolites Produced in Response to Abiotic Stresses Has Potential Application in Pharmaceutical Product Development. Molecules.

[7] Gutiérrez-Grijalva E.P., López-Martínez L.X., Contreras-Angulo L.A., Elizalde-Romero C.A., Heredia J.B. (2020). Plant Alkaloids: Structures and Bioactive Properties. Plant-Derived Bioactives.

[8] Mazid M., Khan T.A., Mohammad F. (2011). Role of Secondary Metabolites in Defense Mechanisms of Plants. Biol. Med..

[9] Thawabteh A., Juma S., Bader M., Karaman D., Scrano L., Bufo S., Karaman R. (2019). The Biological Activity of Natural Alkaloids against Herbivores, Cancerous Cells and Pathogens. Toxins.

[10] Youssef F.S., Simal-Gandara J. (2021). Comprehensive Overview on the Chemistry and Biological Activities of Selected Alkaloid Producing Marine-Derived Fungi as a Valuable Reservoir of Drug Entities. Biomedicines.

[11] Hashim S.E., Basar N., Abd Samad A., Jamil S., Bakar M.B., Jamalis J., Abd-Aziz N., Wagiran A., Razak M.R.M.A., Samad A.F.A. (2024). Advancing Alkaloid-Based Medicines: Medical Applications, Scalable Production and Synthetic Innovations. Phytochem. Rev..

[12] Wu Y., Ren D., Gao C., Li J., Du B., Wang Z., Qian S. (2023). Recent Advances for Alkaloids as Botanical Pesticides for Use in Organic Agriculture. Int. J. Pest. Manag..

[13] Bhambhani S., Kondhare K.R., Giri A.P. (2021). Diversity in Chemical Structures and Biological Properties of Plant Alkaloids. Molecules.

[14] Yahyazadeh M., Nowak M., Kima H., Selmar D. (2017). Horizontal Natural Product Transfer: A Potential Source of Alkaloidal Contaminants in Phytopharmaceuticals. Phytomedicine.

[15] Yang G.Z., Zhu J.K., Yin X.D., Yan Y.F., Wang Y.L., Shang X.F., Liu Y.Q., Zhao Z.M., Peng J.W., Liu H. (2019). Design, Synthesis, and Antifungal Evaluation of Novel Quinoline Derivatives Inspired from Natural Quinine Alkaloids. J. Agric. Food Chem..

[16] Antika L.D., Triana D., Ernawati T. (2020). Antimicrobial Activity of Quinine Derivatives against Human Pathogenic Bacteria. IOP Conf. Ser. Earth Environ. Sci..

[17] Stork G., Niu D., Fujimoto A., Koft E.R., Balkovec J.M., Tata J.R., Dake G.R. (2001). The First Stereoselective Total Synthesis of Quinine. J. Am. Chem. Soc..

[18] Shiomi S., Misaka R., Kaneko M., Ishikawa H. (2019). Enantioselective Total Synthesis of the Unnatural Enantiomer of Quinine. Chem. Sci..

[19] Nair V., Menon R.S., Vellalath S. (2006). Asymmetric Synthesis of Quinine: A Landmark in Organic Synthesis. Nat. Prod. Comm..

[20] O’Donovan D.H., Aillard P., Berger M., de la Torre A., Petkova D., Knittl-Frank C., Geerdink D., Kaiser M., Maulide N. (2018). C−H Activation Enables a Concise Total Synthesis of Quinine and Analogues with Enhanced Antimalarial Activity. Angew. Chem. Int. Ed..

[21] Lee J., Chen D.Y.-K. (2019). A Local-Desymmetrization-Based Divergent Synthesis of Quinine and Quinidine. Angew. Chem. Int. Ed..

[22] Jiang Y., Deiana L., Zhang K., Lin S., Córdova A. (2019). Total Asymmetric Synthesis of Quinine, Quinidine, and Analogues via Catalytic Enantioselective Cascade Transformations. Eur. J. Org. Chem..

[23] Cagir A., Jones S.H., Gao R., Eisenhauer B.M., Hecht S.M. (2003). Luotonin A. A naturally occurring human DNA topoisomerase I poison. J. Am. Chem. Soc..

[24] Yang G.Z., Zhang J., Peng J.W., Zhang Z.J., Zhao W.B., Wang R.X., Ma K.Y., Li J.C., Liu Y.Q., Zhao Z.M. (2020). Discovery of Luotonin A Analogues as Potent Fungicides and Insecticides: Design, Synthesis and Biological Evaluation Inspired by Natural Alkaloid. Eur. J. Med. Chem..

[25] Kwon S.H., Seo H.A., Cheon C.H. (2016). Total Synthesis of Luotonin A and Rutaecarpine from an Aldimine via the Designed Cyclization. Org. Lett..

[26] Baguia H., Deldaele C., Romero E., Michelet B., Evano G. (2018). Copper-Catalyzed Photoinduced Radical Domino Cyclization of Ynamides and Cyanamides: A Unified Entry to Rosettacin, Luotonin A, and Deoxyvasicinone. Synthesis.

[27] Maiti M., Nandi R., Chaudhuri K. (1983). The effect of ph on the absorption and fluorescence spectra of sanguinarine. Photochem. Photobiol..

[28] Croaker A., King G.J., Pyne J.H., Anoopkumar-Dukie S., Liu L. (2016). Sanguinaria Canadensis: Traditional Medicine, Phytochemical Composition, Biological Activities and Current Uses. Int. J. Mol. Sci..

[29] Zhao Z.M., Shang X.F., Lawoe R.K., Liu Y.Q., Zhou R., Sun Y., Yan Y.F., Li J.C., Yang G.Z., Yang C.J. (2019). Anti-Phytopathogenic Activity and the Possible Mechanisms of Action of Isoquinoline Alkaloid Sanguinarine. Pestic. Biochem. Physiol..

[30] Tatton M.R., Simpson I., Donohoe T.J. (2014). New Methods for the Synthesis of Naphthyl Amines; Application to the Synthesis of Dihydrosanguinarine, Sanguinarine, Oxysanguinarine and (±)-Maclekarpines B and C. Chem. Commun..

[31] Parvatkar P.T., Parameswaran P.S. (2016). Indoloquinolines: Possible Biogenesis from Common Indole Precursors and Their Synthesis Using Domino Strategies. Curr. Org. Synth..

[32] Chen Y.J., Liu H., Zhang S.Y., Li H., Ma K.Y., Liu Y.Q., Yin X.D., Zhou R., Yan Y.F., Wang R.X. (2021). Design, Synthesis, and Antifungal Evaluation of Cryptolepine Derivatives against Phytopathogenic Fungi. J. Agric. Food Chem..

[33] Shang X.F., Dai L.X., Zhang Z.J., Yang C.J., Du S.S., Wu T.L., He Y.H., Zhu J.K., Liu Y.Q., Yan Y.F. (2021). Integrated Proteomics and Transcriptomics Analyses Reveals the Possible Antifungal Mechanism of an Indoloquinoline Alkaloid Neocryptolepine against Rhizoctonia Solani. J. Agric. Food Chem..

[34] Ding Y.Y., Jin Y.R., Luo X.F., Zhang S.Y., Dai T.L., Ma L., Zhang Z.J., Wu Z.R., Jin C.X., Liu Y.Q. (2024). Design, Synthesis, and Antimicrobial Activity Evaluation of Novel Isocryptolepine Derivatives against Phytopathogenic Fungi and Bacteria. J. Agric. Food Chem..

[35] Abe T., Suzuki T., Anada M., Matsunaga S., Yamada K. (2017). 2-Hydroxyindoline-3-Triethylammonium Bromide: A Reagent for Formal C3-Electrophilic Reactions of Indoles. Org. Lett..

[36] Mudududdla R., Mohanakrishnan D., Bharate S.S., Vishwakarma R.A., Sahal D., Bharate S.B. (2018). Orally Effective Aminoalkyl 10H-Indolo[3,2-b]Quinoline-11-Carboxamide Kills the Malaria Parasite by Inhibiting Host Hemoglobin Uptake. ChemMedChem.

[37] Majhi B., Ganguly S., Palit S., Parwez A., Saha R., Basu G., Dutta S. (2023). Sequence-Specific Dual DNA Binding Modes and Cytotoxicities of N-6-Functionalized Norcryptotackieine Alkaloids. J. Nat. Prod..

[38] Zhu J.K., Gao J.M., Yang C.J., Shang X.F., Zhao Z.M., Lawoe R.K., Zhou R., Sun Y., Yin X.D., Liu Y.Q. (2020). Design, Synthesis, and Antifungal Evaluation of Neocryptolepine Derivatives against Phytopathogenic Fungi. J. Agric. Food Chem..

[39] Thobokholt E.N., Larghi E.L., Bracca A.B.J., Kaufman T.S. (2020). Isolation and Synthesis of Cryptosanguinolentine (Isocryptolepine), a Naturally-Occurring Bioactive Indoloquinoline Alkaloid. RSC Adv..

[40] Akitake M., Noda S., Miyoshi K., Sonoda M., Tanimori S. (2021). Access to γ-Carbolines: Synthesis of Isocryptolepine. J. Org. Chem..

[41] Olmedo G.M., Cerioni L., González M.M., Cabrerizo F.M., Rapisarda V.A., Volentini S.I. (2017). Antifungal Activity of β-Carbolines on Penicillium Digitatum and Botrytis Cinerea. Food Microbiol..

[42] Kuang Y., Huang J., Chen F. (2006). Practical and Phase Transfer–Catalyzed Synthesis of 6-Methoxytryptamine. Synth. Commun..

[43] Du H., Tian S., Chen J., Gu H., Li N., Wang J. (2016). Synthesis and Biological Evaluation of N9-Substituted Harmine Derivatives as Potential Anticancer Agents. Bioorg Med. Chem. Lett..

[44] Liu W., Liu X., Tian L., Gao Y., Liu W., Chen H., Jiang X., Xu Z., Ding H., Zhao Q. (2021). Design, Synthesis and Biological Evaluation of Harmine Derivatives as Potent GSK-3β/DYRK1A Dual Inhibitors for the Treatment of Alzheimer’s Disease. Eur. J. Med. Chem..

[45] Sathiyalingam S., Roesner S. (2022). Synthesis of A- and Β-Carbolines by a Metalation/Negishi Cross-Coupling/S N Ar Reaction Sequence. Adv. Synth. Catal..

[46] Zhang P., Sun X., Xu B., Bijian K., Wan S., Li G., Alaoui-Jamali M., Jiang T. (2011). Total Synthesis and Bioactivity of the Marine Alkaloid Pityriacitrin and Some of Its Derivatives. Eur. J. Med. Chem..

[47] Wang T.N., Yang S., Shi S.Y., Yuan W.Y., Chen J.X., Duan Z.Y., Lu A.D., Wang Z.W., Wang Q.M. (2021). Pityriacitrin Marine Alkaloids as Novel Antiviral and Anti-Phytopathogenic-Fungus Agents. Pest. Manag. Sci..

[48] Zhu Y., Liu M., Cai Q., Jia F., Wu A. (2013). A Cascade Coupling Strategy for One-Pot Total Synthesis of Β-Carboline and Isoquinoline-Containing Natural Products and Derivatives. Chem.—Eur. J..

[49] Huang D., Zhang Z., Li Y., Liu F., Huang W., Min Y., Wang K., Yang J., Cao C., Gong Y. (2022). Carboline Derivatives Based on Natural Pityriacitrin as Potential Antifungal Agents. Phytochem. Lett..

[50] Franco L.H., Joffé E.B.D.K., Puricelli L., Tatian M., Seldes A.M., Palermo J.A. (1998). Indole Alkaloids from the Tunicate *Aplidium meridianum*. J. Nat. Prod..

[51] Dong J., Huang S.S., Hao Y.N., Wang Z.W., Liu Y.X., Li Y.Q., Wang Q.M. (2020). Marine-Natural-Products for Biocides Development: First Discovery of Meridianin Alkaloids as Antiviral and Anti-Phytopathogenic-Fungus Agents. Pest. Manag. Sci..

[52] Xiao L. (2022). A Review: Meridianins and Meridianins Derivatives. Molecules.

[53] Jiang B., Yang C.G. (2000). Synthesis of Indolylpyrimidines via Cross-Coupling of Indolylboronic Acid with Chloropyrimidines: Facile Synthesis of Meridianin D. Heterocycles.

[54] Fresneda P.M., Molina P., Bleda J.A. (2001). Synthesis of the Indole Alkaloids Meridianins from the Tunicate Aplidium Meridianum. Tetrahedron.

[55] Karpov A.S., Merkul E., Rominger F., Müller T.J.J. (2005). Concise Syntheses of Meridianins by Carbonylative Alkynylation and a Four-Component Pyrimidine Synthesis. Angew. Chem. Int. Ed..

[56] Linares-Otoya L., Liu Y., Linares-Otoya V., Armas-Mantilla L., Crüsemann M., Ganoza-Yupanqui M.L., Campos-Florian J., König G.M., Schäberle T.F. (2019). Biosynthetic Basis for Structural Diversity of Aminophenylpyrrole-Derived Alkaloids. ACS Chem. Biol..

[57] Kilani J., Fillinger S. (2016). Phenylpyrroles: 30 Years, Two Molecules and (Nearly) No Resistance. Front. Microbiol..

[58] Pawar S., Chaudhari A., Prabha R., Shukla R., Singh D.P. (2019). Microbial Pyrrolnitrin: Natural Metabolite with Immense Practical Utility. Biomolecules.

[59] Nakano H., Umio S., Kariyone K., Tanaka K., Kishimoto T., Noguchi H., Ueda I., Nakamura H., Morimoto T. (1966). Total Synthesis of Pyrrolnitrin, a New Antibiotic. Tetrahedron Lett..

[60] Morrison M.D., Hanthorn J.J., Pratt D.A. (2009). Synthesis of Pyrrolnitrin and Related Halogenated Phenylpyrroles. Org. Lett..

[61] Bray B.L., Mathies P.H., Naef R., Solas D.R., Tidwell T.T., Artis D.R., Muchowski J.M. (1990). N-(Triisopropylsilyl)Pyrrole. A Progenitor “Par Excellence” of 3-Substituted Pyrroles. J. Org. Chem..

[62] Li C.-S., An C.-Y., Li X.-M., Gao S.-S., Cui C.-M., Sun H.-F., Wang B.-G. (2011). Triazole and Dihydroimidazole Alkaloids from the Marine Sediment-Derived Fungus *Penicillium Paneum* SD-44. J. Nat. Prod..

[63] Reddy A.S., Reddy V.K., Rao G.N., Basavaiah K. (2022). An Efficient Total Synthesis of a Natural Penipanoid A. Tetrahedron Lett..

[64] Reddy A.S., Reddy V.K., Rao G.N., Basavaiah K., Nagulapalli Venkata K.C. (2023). A Novel and Efficient One-Pot Strategy for the Synthesis of 1,2,4-Triazoles: Access to Synthesis of Penipanoid A and Its Analogues. RSC Adv..

[65] Das J., Bhan A., Mandal S.S., Lovely C.J. (2013). Total Syntheses and Cytotoxicity of Kealiiquinone, 2-Deoxy-2-Aminokealiiquinone and Analogs. Bioorg Med. Chem. Lett..

[66] Li G., Guo J., Wang Z., Liu Y., Song H., Wang Q. (2018). Marine Natural Products for Drug Discovery: First Discovery of Kealiinines A-C and Their Derivatives as Novel Antiviral and Antiphytopathogenic Fungus Agents. J. Agric. Food Chem..

[67] Das J., Koswatta P.B., Jones J.D., Yousufuddin M., Lovely C.J. (2012). Total Syntheses of Kealiinines A–C. Org. Lett..

[68] Guo P., Li G., Liu Y., Lu A., Wang Z., Wang Q. (2018). Naamines and Naamidines as Novel Agents against a Plant Virus and Phytopathogenic Fungi. Mar. Drugs.

[69] Ohta S., Tsuno N., Nakamura S., Taguchi N., Yamashita M., Kawasaki I., Fujieda M. (2000). Total Syntheses of Naamine A and Naamidine A, Marine Imidazole Alkaloids. Heterocycles-Sendai Inst. Heterocycl. Chem..

[70] Kawasaki I., Nakamura S., Yanagitani S., Kakuno A., Yamashita M., Ohta S. (2001). New Access to 1,3-Dialkyl-2,3-Dihydro-2-Imino-1H-Imidazoles and Their Application to the First Total Synthesis of Naamine B, a Marine 2,3-Dihydro-2-Imino-1,3-Dimethyl-1H-Imidazole Alkaloid. J. Chem. Soc. Perkin Trans..

[71] Ermolat’ev D.S., Bariwal J.B., Steenackers H.P.L., De Keersmaecker S.C.J., Van der Eycken E.V. (2010). Concise and Diversity-Oriented Route toward Polysubstituted 2-Aminoimidazole Alkaloids and Their Analogues. Angew. Chem..

[72] Koswatta P.B., Lovely C.J. (2010). Total Syntheses of Naamidine G and 14-Methoxynaamidine G. Tetrahedron Lett..

[73] Koswatta P.B., Lovely C.J. (2010). Concise Total Synthesis of Naamine G and Naamidine H. Chem. Commun..

[74] Xin Z., OuYang Q., Wan C., Che J., Li L., Chen J., Tao N. (2019). Isolation of Antofine from Cynanchum Atratum BUNGE (Asclepiadaceae) and Its Antifungal Activity against Penicillium Digitatum. Postharvest. Biol. Technol..

[75] Oh J., Kim G.D., Kim S., Lee S.K. (2017). Antofine, a Natural Phenanthroindolizidine Alkaloid, Suppresses Angiogenesis via Regulation of AKT/MTOR and AMPK Pathway in Endothelial Cells and Endothelial Progenitor Cells Derived from Mouse Embryonic Stem Cells. Food Chem. Toxicol..

[76] Fan Y., Jiang Y., Liu J., Kang Y., Li R., Wang J. (2016). The Anti-Tumor Activity and Mechanism of Alkaloids from Aconitum Szechenyianum Gay. Bioorg Med. Chem. Lett..

[77] Liu Y., Cui Y., Lu L., Gong Y., Han W., Piao G. (2020). Natural Indole-containing Alkaloids and Their Antibacterial Activities. Arch. Pharm..

[78] Wang M., Zhang W., Wang J., Lu J., Huo Y. (2023). Extraction Separation and Antifungal Activities of Cynanchum Komarovii Al. Iljinski. World Sci. Res. J..

[79] Chen C., Qi W., Peng X., Chen J., Wan C. (2019). Inhibitory Effect of 7-Demethoxytylophorine on Penicillium Italicum and Its Possible Mechanism. Microorganisms.

[80] Yi M., Gu P., Kang X.Y., Sun J., Li R., Li X.Q. (2014). Total Synthesis of (±)-Antofine. Tetrahedron Lett..

[81] Ansari A., Ramapanicker R. (2018). Enantioselective Synthesis of (R)-Antofine and (R)-Cryptopleurine. ChemistrySelect.

[82] El-Gendy M.M.A., Shaaban M., Shaaban K.A., El-Bondkly A.M., Laatsch H. (2008). Essramycin: A First Triazolopyrimidine Antibiotic Isolated from Nature^†^. J. Antibiot..

[83] Battaglia U., Moody C.J. (2010). A Short Synthesis of the Triazolopyrimidine Antibiotic Essramycin. J. Nat. Prod..

[84] Abu-Hashem A.A., Gouda M.A. (2017). Synthesis and Antimicrobial Activity of Some Novel Quinoline, Chromene, Pyrazole Derivatives Bearing Triazolopyrimidine Moiety. J. Heterocycl. Chem..

[85] Tee E.H.L., Karoli T., Ramu S., Huang J.X., Butler M.S., Cooper M.A. (2010). Synthesis of Essramycin and Comparison of Its Antibacterial Activity. J. Nat. Prod..

[86] Wang T., Yang S., Li H., Lu A., Wang Z., Yao Y., Wang Q. (2020). Discovery, Structural Optimization, and Mode of Action of Essramycin Alkaloid and Its Derivatives as Anti-Tobacco Mosaic Virus and Anti-Phytopathogenic Fungus Agents. J. Agric. Food Chem..

[87] Shtark O.Y., Shaposhnikov A.I., Kravchenko L.V. (2003). The Production of Antifungal Metabolites by Pseudomonas Chlororaphis Grown on Different Nutrient Sources. Microbiology.

[88] Niu J., Chen J., Xu Z., Zhu X., Wu Q., Li J. (2016). Synthesis and Bioactivities of Amino Acid Ester Conjugates of Phenazine-1-Carboxylic Acid. Bioorg Med. Chem. Lett..

[89] Xiong Y., Zhu X., Hu J., Wang Y., Du X., Li J., Wu Q. (2021). Effect of Introducing Amino Acids into Phenazine-1-Carboxylic Acid on Phloem Mobility. Nat. Prod. Res..

[90] Zhong Y., Liu J., Cheng X., Zhang H., Zhang C., Xia Z., Wu Z., Zhang L., Zheng Y., Gao Z. (2021). Design, synthesis and biological evaluations of diverse Michael acceptor-based phenazine hybrid molecules as TrxR1 inhibitors. Bioorg Chem..

[91] Lv P., Chen Y., Shi T., Wu X., Li Q.X., Hua R. (2018). Synthesis and fungicidal activities of sanguinarine derivatives. Pestic. Biochem. Physiol..

